# Efficacy of NKG2D CAR-T cells with IL-15/IL-15Rα signaling for treating Epstein-Barr virus-associated lymphoproliferative disorder

**DOI:** 10.1186/s40164-024-00553-z

**Published:** 2024-08-19

**Authors:** Qiusui Mai, Bailin He, Shikai Deng, Qing Zeng, Yanwen Xu, Cong Wang, Yunyi Pang, Sheng Zhang, Jinfeng Li, Jinfeng Zeng, Liqin Huang, Yongshui Fu, Chengyao Li, Tingting Li, Xiaojun Xu, Ling Zhang

**Affiliations:** 1 Department of Blood Transfusion, The Seventh Affiliated Hospital, Sun Yat-sen University, Shenzhen, 518107 China; 2 Department of Transfusion Medicine, School of Laboratory Medicine and Biotechnology, Southern Medical University, Guangzhou, 510515 China; 3 Department of Hematology, Nanfang Hospital, Southern Medical Universit, Guangzhou, 510515 China; 4 Department of Obstetrics, He Xian Memorial Affiliated Hospital of Southern Medical University, Guangzhou, 511402 China; 5 Guangzhou Bai Rui Kang (BRK) Biological Science and Technology Limited Company, Guangzhou, 510555 China; 6 Department of Obstetrics, Nanfang Hospital, Southern Medical University, Guangzhou, 510515 China; 7 Shenzhen Bao’an District Central Blood Station, Shenzhen, 518101 China; 8 Shenzhen Blood Center, Shenzhen, 518035 China; 9 Guangzhou Blood Center, Guangzhou, 510095 China

**Keywords:** NKG2D, IL-15/IL-15Rα, CAR-T cells, EBV infection, Post-transplantation

## Abstract

Epstein-Barr virus (EBV) related post-transplant lymphoproliferative disorder (EBV-PTLD) is a life-threatening complication after hematopoietic stem cell transplantation (HSCT) or solid organ transplantation (SOT), for which no standard therapeutic means have been developed. Significant increase expression of natural killer group 2 member D ligands (NKG2DLs) was observed on B-lymphoblastoid cells of EBV-PTLD, indicating NKG2DLs as potential therapeutic targets for treatment of EBV-PTLD. In this study, the recombinant constructs of NKG2D CAR and IL-15/IL-15Rα-NKG2D CAR were generated with a retroviral vector and then transduced to human T cells to produce NKG2D CAR-T and IL-15/IL-15Rα-NKG2D CAR-T cells, respectively. B-lymphoblastoid cell lines (B-LCLs) and the xenografted mouse models were established to evaluate the efficacy of these CAR-T cells. IL-15/IL-15Rα-NKG2D CAR-T cells exhibited superior proliferation and antigen-specific cytotoxic effect compared to NKG2D CAR-T, as IL-15/IL-15Rα signaling promoted the expansion of less differentiated central memory T cells (T_CM_) and increased expression of CD107a and IFN-γ. Moreover, EBV DNA load was dramatically reduced, and 80% B-LCL cells were eliminated by IL-15/IL-15Rα-NKG2D CAR-T cells after co-culturing. In-vivo study confirmed that IL-15/IL-15Rα-NKG2D CAR-T cell therapy significantly enhanced antiviral efficacy in mice, as the serum load of EBV after IL-15/IL-15Rα-NKG2D CAR-T cell infusion was 1500 times lower than the untreated control (*P* < 0.001). The enhanced efficacy of IL-15/IL-15Rα-NKG2D CAR T cells was probably due to the IL-15/IL-15Rα signaling improved homing and persistence of NKG2D CAR-T cells in vivo, and increased the production of IFN-γ, Perforin, and Granulysin. In conclusion, NKG2D CAR-T cells co-expressing IL-15/IL-15Rα promoted the central memory CAR T cell proliferation and improved the homing and persistence of CAR T cells in vivo, resulting in enhanced anti-tumor and anti-viral effects in treating EBV-PTLD.

**Supplementary Information:**

The online version contains supplementary material available at 10.1186/s40164-024-00553-z.

## Background

The Epstein-Barr virus (EBV) belongs to the human γ-herpesvirus family and is also referred as human herpes virus 4 (HHV4) [1, 2]. Approximately 95% of adults worldwide are seropositive to EBV (VCA-IgG^+^) [3]. Following primary infection, EBV replication is regulated by the host immune system, resulting in long-term latent infection in human B lymphocytes. This benign equilibrium is disrupted when the host’s immune function is compromised or certain triggers occur, leading to various EBV-related diseases. EBV infections are categorized into two states: lytic infection and latent infection. The viral products during latent infection are associated with malignant cell transformation, contributing to various B lymphocyte- and epithelial cell-derived malignancies. Furthermore, some viral proteins expressed during latent infection exhibit weaker immunogenicity compared to those expressed during lytic infection, posing challenges for treatment [1, 4]. EBV related post-transplant lymphoproliferative disorder (EBV-PTLD) is a status of EBV latent infection, and a life-threatening complication after hematopoietic stem cell transplantation (HSCT) or solid organ transplantation (SOT) [5, 6]. The conventional treatment for EBV-PTLD includes reducing immunosuppressive agents, administering chemotherapy, CD20 monoclonal antibody/Rituximab and antiviral drugs like ganciclovir and EBV-specific gamma globulin, etc., which often accompany with various side effects [7–9]. Adoptive transfusion of EBV-specific T cells (EBV-CTL) is a cellular therapy that has attracted much attention in past years [10, 11]. Despite many studies have confirmed the efficacy and safety of EBV-CTL in PTLD treatment, the existing limitations hamper its widely used in PTLD, such as immune escape induced by down-regulation of human leukocyte antigen (HLA) I molecule and needing of donors with positive anti-EBV IgG serotypes [12–14].

Currently, there have been reports on chimeric antigen receptor (CAR)-T targeting EBV antigens, such as gp350 [15], EBNA3C [16] and LMP-1 [17], and in clinical practice, CAR-T therapy targeting B cell markers (CD19, CD20, and CD22) have also been reported for the treatment of EBV-PTLD, whose applications are limited by the issues of antigen escape and B-cell lineage immune suppression [18–20]. The physicians must weigh the benefit against the potential for adverse events, and there is still room for the development of new treatments that can combine efficacy with long-term safety and meet patients’ satisfaction (Supplementary Table 1). The Natural killer group 2 member D receptor (NKG2D) acts as a promising role for specific targeting [21, 22]. Normally, the expression of NKG2D ligands (NKG2DLs) is typically absent or low in normal cells; during states of cellular stress such as inflammation, carcinogenesis, and viral invasion, there is a significant upregulation of NKG2DL expression [23, 24]. Different variants of NKG2D-based CAR have been developed, and extensive in vivo mechanistic studies have been conducted to demonstrate the crucial role of cytotoxicity and cytokines in the efficacy of NKG2D CAR adoptive T cell therapy [25–28]. Attractively, significantly increasing of NKG2DLs was observed on B-lymphoblastoid cell line (B-LCL) of EBV-PTLD [29], indicating their potential as therapeutic targets for EBV-PTLD treatment by NKG2D CAR-T cells.

Although studies have demonstrated the significant efficacy of NKG2D CAR-T in the treatment of ovarian cancer [25], triple-negative breast cancer [26], multiple myeloma(MM) [27] and osteosarcoma [28], it is plagued by issues pertaining to limited expansion and persistence [30, 31]. In this study, we demonstrated that the combination of IL-15/IL-15Rα and NKG2D CAR can significantly augment both the effectiveness and safety profiles of CAR-T therapy. IL-15/IL-15Rα is the compounded form of IL-15 and soluble IL-15 receptor α, which can also bind the IL-15 receptor β and γ on immune cells like T cells and NK cells, presenting better stability and safety during action compared with IL-15 [32–35]. The half-life of IL-15/IL-15Rα can be extended from the original 30 min to 20 h, and the biological activity can be increased by at least 50 times, so it is referred as IL-5/IL-15Ra superagonist complex. Clinical [36, 37] and preclinical [38, 39] studies have shown that IL-5/IL-15Ra enhances the efficacy and safety of CAR therapy in cancers compared with IL-15. The structure of IL-15 cytokine exhibits high homology compared with IL-2, belonging to the helical cytokine family. It activates NK, NKT, and CD8^+^ T cells effectively while prolonging T cell lifespan. Unlike IL-2, IL-15 does not initiate activation induced cell death (AICD) or promote Treg proliferation. Additionally, IL-15 exerts stronger activation effects on maintenance of CD8^+^CD44^hi^ memory T cells and immature NK cells (CD56^bright^ NK) compared to IL-2, thus playing a crucial role in antiviral infection and tumor cytotoxicity [40, 41].

Based on the effects of NKG2D CAR and IL-15/IL-15Rα on virus-infected cells and tumor cells, as well as the NKG2DL expressions on B-LCL cells of EBV-PTLD mouse models, we propose IL-15/IL-15Rα co-expressing NKG2D CAR-T for the treatment of EBV PTLD - an approach that has not been reported thus far. In this study, we conducted phenotypic studies on both NKG2D (N) CAR-T and IL-15/IL-15Rα-NKG2D (N15) CAR-T cells, and then explored their anti-tumor and antiviral efficacy in vitro and in vivo in comparison with non-transduced (NT)-T cell controls, aiming at investigating a novel potential approach for the treatment of EBV-PTLD patients.

## Materials and methods

### PBMCs and B-LCLs

Human peripheral blood mononuclear cells (PBMCs) were isolated by density gradient centrifugation from the “Buffy Coat” of healthy blood donors at Guangzhou Blood Center using Human Peripheral Blood Lymphocyte Separation Solution (Tianjin Haoyang Biological Manufacture Co., Ltd, China) (Supplementary Methods). EBV-immortalized B-lymphoblastoid cell Lines (B-LCLs) were transformed from the culture of PBMCs with EBV (the culture supernatant of B95-8 cells, EBV-transformed marmoset B-lymphoblastoid cell line, provided by Dr. Xuan Yi, Department of Infectious Diseases, Southern Hospital, China) according to previously reported protocol [42–44]. The B-LCL cell lines were characterized for CD19 expression and EBV DNA load before they were used in the study (Supplementary Methods and Supplementary Fig. 1). Human PBMCs for the use in this study have been approved by the Ethics Committee of Guangzhou Blood Center, Guangzhou China (Approval No. 2022-068).

The NKG2DL markers (MICA, MICA/B, ULBP-1, ULBP-3) on B-LCLs were detected by flow cytometry with a panel of antibodies (Supplementary Table 2). EBV load was measured by real-time quantitative PCR (qPCR) according to the protocol of EBV nucleic acid quantitative detection kit (Beijing Gene Technology Co., Ltd., China) (Supplementary Methods).

### Recombinant retroviruses and CAR-T cells

The constructs of NKG2D (N) CAR and IL-15/IL-15Rα-NKG2D (N15) CAR consists of signal peptide, NKG2D ectodomain (aa 82–216), CD8 TM, cytoplasmic signaling domains from 4-1BB and CD3ζ, or plus IL-15/IL-15Rα (with His-tag). These gene fragments were synthesized by Huada Gene Co., Ltd. (Shenzhen, China) and inserted into the retroviral vector plasmid pSFG.eGFP (MiaoLingBio, Wuhan, Hubei, China) to get pSFG-N CAR (NKG2D CAR) and pSFG-N15 CAR (IL-15/IL-15Rα-NKG2D CAR) (Supplementary Fig. 2), which were verified by qPCR, restriction enzyme digestion, sequencing and Western blot analysis with specific antibodies (Supplementary Methods, Supplementary Table 2, Supplementary Figs. 3 and 4). Recombinant retroviruses were produced by co-transfection of retroviral plasmids and packing plasmids (PegPam3 plasmid and RDF plasmid) into 293T cells as previous described [45, 46].

To generate CAR-T cells, the PBMCs were seeded at 2 × 10^6^ cells per well in a 24-well plate in 1 ml of T cell serum-free culture medium (ExCell Biology, Inc., Shanghai, China) supplemented with 100IU/ml IL-2 and stimulated with 5 µl per well of anti-CD3/CD28 beads (Miltenyi Biotec, Germany) for 2 days. A RetroNectin^®^ (T100A, TaKaRa)-coated plate was prepared at the concentration of 7 µg/ml retronectin in 1 ml PBS per well and incubated overnight at 4℃. Before transduction, the retronectin supernatant was removed and 2 ml of retrovirus supernatant were pooled in the well. Then the plate was sealed and centrifuged at 2000 g for 90 min at room temperature to get retrovirus-coated plate. Finally, half a million of stimulated T cells were seed in T cell culture medium with 100IU/ml IL-2 and cultured at 37 °C in a humidified 5% CO2 incubator for more than 72 h before harvested. The recombinant N or N15 CAR retrovirus transduced T cells were designated as N or N15 CAR-T cells, and the non-transduced (NT)-T cells were used as controls. The expression of NKG2D CAR was detected by flow cytometry with anti-human CD314 (NKG2D) antibody (320808, Biolegend, Japan) (Supplementary Methods). The secretion of IL-15/IL-15Rα was measured with Human IL-15 ELISA Kit (MultiSciences, Zhejiang, China) (Supplementary Methods). Total numbers of PBMCs, B-LCLs and T cells were calculated by a cell counter using trypan blue staining.

### Cell viability and proliferation detection

The CAR-T cell viability was detected with LIVE/DEAD Fixable Dead Cell Staining kit (LIVE/DEAD™ Fixable Near IR Viability Kit, L34992, Thermo, USA), and the proliferation was measured by flow cytometry with Flow cytometry Absolute Counting Beads kit according to the instructions (CountBright absolute counting beads, C36950, Invitrogen, USA). Flow cytometric analysis was performed on a BD LSRFortessa (BD Biosciences, USA) at the Southern Medical University Central Laboratory, and the data were analyzed with FlowJo v10 (Supplementary Methods and Supplementary Fig. 5). The equipment has been calibrated regularly.

### Phenotyping and RNA-seq analysis

The phenotypes of NT-T, N and N15 CAR-T cell groups, including CD4^+^/CD8^+^ ratio, naïve/memory (CD45RA/CCR7), specific activation (CD25 and CD69), and exhaustion markers (PD-1, LAG-3 and TIM-3), were detected by flow cytometry with specific antibodies (Supplementary Table 2).

The transcriptome samples of NT-T, N and N15 CAR-T cells were sequenced commercially in by Guangzhou Huayin Laboratory Center (Guangzhou, China), of which the procedure was demonstrated in Supplementary Methods. The Principal Component Analysis (PCA), Gene Ontology (GO), Kyoto Encyclopedia of Genes and Genomes (KEGG) pathway enrichment analysis and Gene Set Enrichment Analysis (GSEA) were performed.

### Cytotoxicity assay

B-LCLs as target cells were co-cultured with three groups of NT-T, N and N15 CAR-T cells, and stained with Cell Proliferation Dye eFluor 450 (BV450, 65-0842-85, ThermoFisher, USA). The lysis of B-LCLs was quantified at corresponding time points with the method of flow cytometric cytotoxicity assay as previous reported [47]. All cells in each assay were collected and stained with the fluorochrome-labeled monoclonal antibodies LIVE/DEAD Fixable Dead Cell Staining (APC-Cy7, L34992, Thermo, USA) and PE-Cy7 anti-human CD3 (300420, Biolegend, Japan). Gating strategies were illustrated in Supplementary Fig. 6. Finally, the number of live B-LCLs (CD3^−^APC-Cy7^−^BV450^+^) were counted by flow cytometry and the percentage of lysis was calculated according to the following formula:

$$\:{\text{Lysis}}\:\left( \% \right) = \frac{{\left( \begin{gathered}{\text{Control}}\:{\text{group}}\:{\text{B}} - {\text{LCL}}\:{\text{count}} - \hfill \\{\text{Experimental}}\:{\text{group}}\:{\text{B}} - {\text{LCL}}\:{\text{count}} \hfill \\ \end{gathered} \right)}}{{{\text{Control}}\:{\text{gounp}}\:{\text{B}} - {\text{LCL}}\:{\text{count}}}} \times \:100\%$$

### Detection of in vitro cytokines

The expression of CD107a, IFN-γ, TNF-α, IL-2 or Granzyme B was detected by degranulation assay and intracellular staining (ICS) in the co-cultures of B-LCLs or PBMCs with NT-T, N and N15 CAR-T cells (E: T = 5 × 10^5^ T cells/15 × 10^5^ B-LCLs) for 5 hours, respectively (Supplementary Methods). Levels of IL-15, IL-2, IFN-γ, TNF-α, IL-6, IL-10 and GM-CSF release were monitored in the co-cultures at 6, 24, 48 and 72 h by ELISA (Supplementary Methods). Antibodies and ELISA kits used were listed in Supplementary Table 2.

### Establishment of EBV-PTLD mouse models by B-LCL xenografted mice

The 6–8 weeks old Non-obese diabetic (NOD) Cg-Prkdc^scid^ IL2rg^tm1Wjl^/SzJ (NSG) mice were purchased from Jinwei Biotech Co. Ltd., Guangzhou, China. Twelve mice of each group were intravenously injected by a million B-LCLs to generate 4 groups of xenograft mouse models, mimicking EBV-PTLD for testing of therapeutic effects by NT-T, N and N15 CAR-T cells in comparison with B-LCL mice (tumor bearing treated with PBS, *n* = 12). Naïve NGS mice (non-tumor bearing treated with PBS, *n* = 12) were used as normal controls (NC). Three days after the B-LCL injection, 5 millions of NT-T, N or N15 CAR-T cells were injected into the tail vein of mice in each group, respectively. The mice of each group were randomly divided into two parts, the first part mice (*n* = 30/5 groups) were used to observe survival time and body weight changes, the second part mice (*n* = 30/5 groups) were used to measure EBV load, tumor burden, magnetic resonance imaging, and T cell counts, etc. Mice that lost more than 30% of body weight were euthanized. Detailed protocol regarding to mice was approved by the Ethics Committee of Southern Medical University (No. SMUL2023080). All procedures with mice were performed in accordance with the relevant requirements by institutional animal care and used Committee of Southern Medical University (Guangzhou, China).

### Micro-magnetic resonance imaging (Micro-MRI)

On Day 5 after T cell treatment, 3 mice of each group from second part mice were selected and anesthetized with isoflurane (oxygen delivered at 0.5 L/min with 3% isoflurane for induction and 1.5% isoflurane for maintenance). Then the mice were imaged with micro-MRI (Pharma Scan 70/16, US) under the fixed parameter (coronal T2WI imaging; magnet strength = 7T; coil diameter = 23 mm; image size: 256 × 256 mm; Echo time: 30.2 ms; Repetition time: 2300 ms; FOV = 30*25; Layer thickness = 0.7 mm) and data was analyzed with Paravision 6.0 software.

### Evaluation of tumor burden and T cell count in blood and tissues

On Day 21 after T cell treatment, six mice of each groups from second part mice were sacrificed by breaking the neck to obtain the samples of peripheral blood, liver, lung, kidney, spleen and bone marrow tissues. The samples were processed by mechanical grinding, and then filtered through 70 μm cell strainer (352350, Falcon) to get single-cell suspension and proceed to Red Blood Cell Lysis (00-4300-54, eBioscience) at 4℃. Finally, the cell suspension was washed and divided into 2 aliquots, for evaluating tumor burden and T cell count by flow cytometry (stained with PerCP Cy5.5 anti-human CD3, PE anti-human CD19; Supplementary Table 2).

### Haematoxylin-eosin (H&E) staining

Some part of spleen samples excised from the mice were fixed in 4% paraformaldehyde and then prepared into 2 μm paraffin sections for examination. The H&E staining for spleen tissue was conducted by Guangzhou Huayin Laboratory Center (Guangzhou, China).

### Measurement of in vivo cytokine release

On the other hand, a customized LEDGENDplex™ Human CD8/NK Panel (13-plex) with V-bottom Plate (741065, BioLegend, Japan) was used to detect the secretion of human IL-2, IL-4, IL-10, IL-6, IL-17 A, TNF-α, sFas, sFasL, IFN-γ, Granzyme A, Granzyme B, Perforin and Granulysin in the plasmas of mice (Supplementary methods). Sample data were analyzed with the Qognit software (https://legendplex.qognit.com/; BioLegend, Japan).

### Statistical analysis

Continuous values are shown as mean ± SD, with n denoting the number of tests. Statistical analysis of the difference among experiment groups was performed by using the One-way ANOVA and multiple comparison with a Bonferroni multiple comparison correction with IBM SPSS Statistics 20.0 and GraphPad Prism 7 software. Kaplan‒Meier curves were used to estimate the survival rates. Differences in statistical analysis are indicated by asterisks: ****P < 0.0001; ***P < 0.001; **P < 0.01; *P < 0.05.

## Results

### Generation and characterization of NKG2D CAR-T cells

**Fig. 1:**
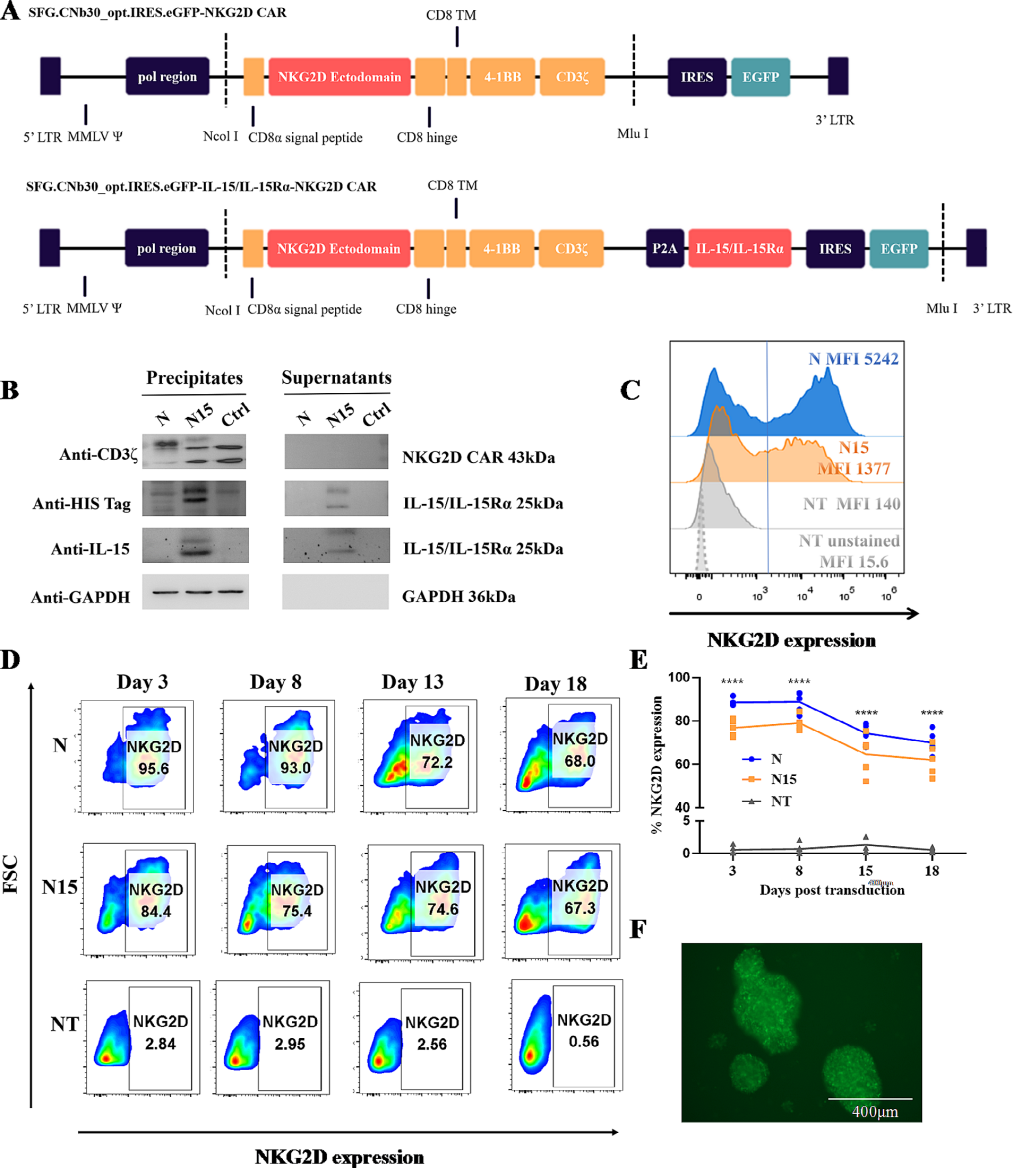
NKG2D CAR and IL-15/IL-15Rα-NKG2D CAR constructs and transduction to T cells. (**A**) Schematic diagram of the NKG2D (N) CAR and IL-15/IL-15Rα-NKG2D (N15) CAR constructs. 5’ LTR or 3’ LTR, long terminal repeat from Moloney murine leukemia virus (MMLV); MMLVΨ, packaging signal of MMLV; pol region, MMLV pol region containing the splice acceptor site; P2A, 2 A peptide from porcine teschovirus-1 polyprotein. (**B**) Identification of NKG2D CAR and IL-15/IL-15Rα expressions in the precipitates and supernatants of 293T cell cultures by Western blot after transduction with three types of retroviruses. N: NKG2D CAR retrovirus transduction; N15: IL-15/IL-15Rα-NKG2D CAR retrovirus transduction; Ctrl: empty retrovirus transduction. (**C**) Representative flow cytometric histograms of N and N15 CAR-T and non-transduced (NT)-T cells stained by anti-NKG2D, and unstained NT-T cell controls. (**D**) Representative flow cytometric scatter plot (FSC) of N and N15 CAR-T cells and NT-T cells on days 3, 8, 13 and 18 post transduction. (**E**) Statistics graph of transduction efficiency of N and N15 CAR-T and NT-T cell groups. Data were presented as the mean of 3 independent tests from 5 individual donors. ****, the values of percentage of NKG2D in both N and N15 were higher than that of NT group (*P* < 0.0001). There is no statistically different between N and N15 group (*P*>0.05). (**F**) General morphology and EGFP expression of CAR-T of N group on Day 8 post transduction (Inverted fluorescence microscope, scale = 400 μm)

The recombinant retrovirus construct of NKG2D (N) CAR contains the core structure of a N CAR fragment (1142 bp), encoding CD8 signal peptide, NKG2D ectodomain, CD8 hinge and CD8 TM, cytoplasmic signaling domains of 4-1BB and CD3ζ (Fig. 1A top panel). At downstream of NKG2D CAR elements, an IL-15/IL-15Rα fragment (687 bp) is linked for encoding IL-15 (342 bp), linker peptide (60 bp) and IL-15Rα sushi domain (with high affinity to IL-15, 285 bp, with His-tag), which is designated as IL-15/IL-15Rα-NKG2D (N15) CAR (Fig. 1A bottom panel). Both N and N15 CAR retroviruses with EGFP fluorescence reporter were packaged and produced in 293T cells. The proteins of NKG2D CAR (43 KDa) and IL-15/IL-15Rα complex (25KDa) were detected by Western blot with anti-human CD3ζ, IL-15 and His-Tag antibodies respectively in 72 h cell cultures from N and N15 CAR transduced 293T cells but not from empty-retrovirus control (Ctrl) transduced cells (Fig. 1B).

T cells were transduced by N or N15 CAR retroviruses, respectively. On N and N15 CAR-T cells or NT-T cells, the expression of NKG2D was measured by flow cytometry. The mean fluorescence intensity (MFI) of anti-NKG2D staining was significantly increased in N and N15 CAR-T cell groups comparing with the base lines of NT-T cell groups (NT vs. unstained NT) (Fig. 1C). The rate of NKG2D CAR expression reached to 95.6% in N CAR-T cell group and 84.4% in N15 CAR-T cell group on Day 3, and then > 65% in both N and N15 CAR-T cell groups on Day 18 post infection (Fig. 1D-F). In addition, the EGFP expression was found consistent with the NKG2D CAR expression, which could be used as a surrogate marker for detection of N and N15 CAR-T cells in the following experiments (Supplementary Fig. 7).

### The effects of addition of IL-15/IL-15Rα module on NKG2D CAR-T cells

**Fig. 2:**
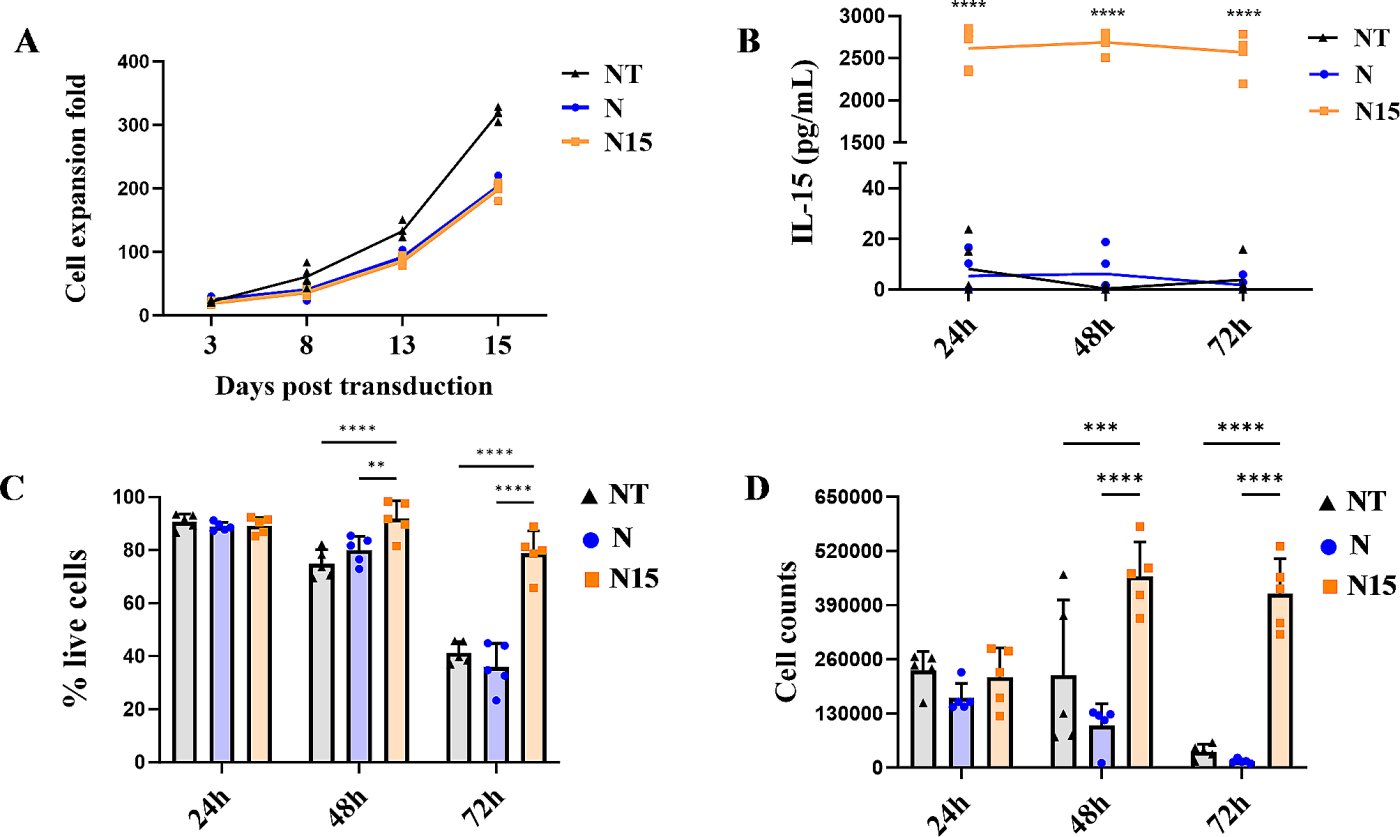
Viability and proliferation of NT-T, N and N15 CAR-T cells. (**A**) Proliferation multiplicity of NT-T, N and N15 CAR-T cells in culturing with IL-2 maintenance. (**B**) Secretion of IL-15/IL-15Rα in cultures without IL-2 maintenance detected by IL-15 ELISA kit. (**C**) The percentage of viable T cells in cultures without IL-2 maintenance. IL-15 concentration in N15 group was higher than that in NT or N group, but no statistically different between NT and N groups (*P*>0.05). (**D**) The numbers of viable T cells in cultures without IL-2 maintenance. The data were presented as the mean of 3 independent tests from 5 individual donors. * *P* < 0.05, ** *P* < 0.01, *** *P* < 0.001, **** *P* < 0.0001

By conventionally T cell culturing with IL-2, the growth curve (fold change) showed that the normal NT-T cell group presented the strongest proliferation ability (*P <* 0.0001), increasing more than 320-folds after 15 days post transduction, while N and N15 CAR-T cell groups proliferated 200-folds approximately but no significant difference between N and N15 CAR-T cell groups (*P* > 0.05) (Fig. 2A).

Mimicking in vivo situation without IL-2 maintenance, 0.5 million of NT-T, N and N15 CAR-T cells were harvested on Day 8 post transduction and cultured in a 48 well plate. The mean concentration of IL-15/IL-15Rα in culture supernatant of N15 CAR-T cell group was detected more than 2500pg/mL, while the lower concentration (< 10pg/ml) was detected in those of NT-T and N CAR-T cell groups (Fig. 2B, Supplementary Table 3). After culturing for 24–72 h, cell viability among NT-T and N CAR-T cell groups was declined from 90% to < 40%, whereas cell viability among N15 CAR-T cell group still maintained 70 ~ 80% viable cells (Fig. 2C). The live cell counts of three groups were determined using the Flow cytometry Absolute Counting Beads simultaneously (Fig. 2D), in which the cell counts from N15 CAR-T cell group were increased approximately twice in 48–72 h cultures than those in 24 h culture (*P* < 0.001), while the cell counts from N CAR-T and NT-T cell groups were largely decreased in 48–72 h cultures.

### Characteristics of NKG2D CAR-T cells

**Fig. 3:**
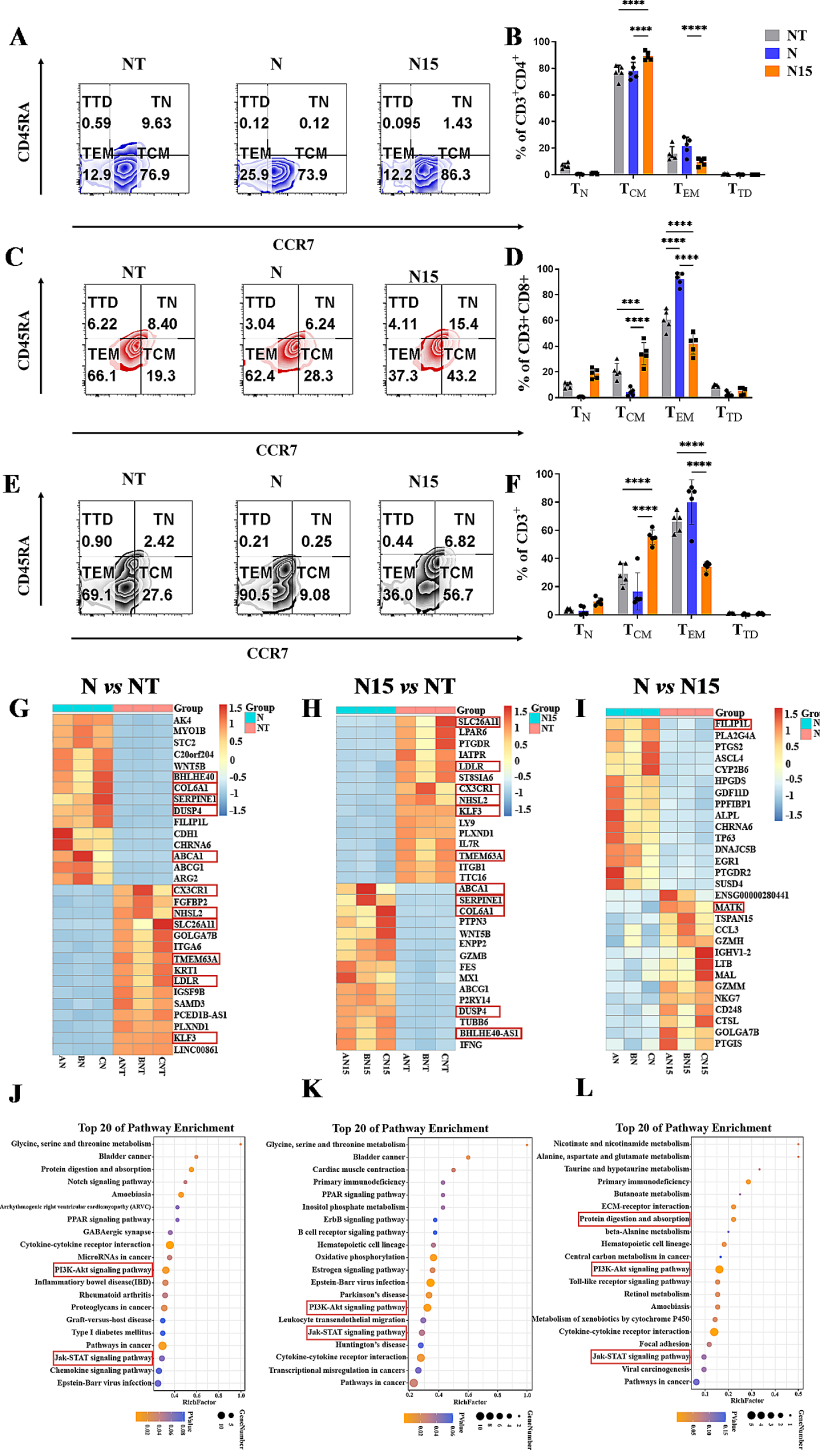
Phenotype and RNA-seq analysis of three types of T cells. (**A**,**B**) Representative flow cytometric analysis (Zeta Plot) for naive/memory T cell subset in CD4^+^ T cells and statistics graph. (**C**,**D**) Representative flow cytometric analysis (Zeta Plot) for naive/memory T cell subset in CD8^+^ T cells and statistics graph. (**E**,**F**) Representative flow cytometric analysis (Zeta Plot) for naive/memory T cell subset in CD3^+^ T cells and statistics graph. T_N_, naive T (CD45RA^+^CCR7^+^); T_CM_, central memory T (CD45RA^−^CCR7^+^); T_EM_, effector memory T (CD45RA^−^CCR7^−^) and T_TD_, terminally differentiated effector T (CD45RA^+^CCR7^−^). The data were presented as the mean of 3 independent tests from 5 individual donors. * *P* < 0.05, ** *P* < 0.01, *** *P* < 0.001, **** *P* < 0.0001. (**G**-**I**) Heatmap of top 15 DEGs ranked by *P* value (the data were obtained from 3 individual donors). (**J**-**K**) Bubble Plot of KEGG pathway analysis (*n* = 3). The genes and pathways highlighted in the red rectangles were selected for possibly associating with N or N15 CAR-T cells

The characteristics of phenotypes were evaluated in culturing of T cells (E: T = 2:1) harvested on Day 13 post transduction. The naive/memory T cell subsets were detected by flow cytometry according to the published article [48], including T_N_ (naive T, CD45RA + CCR7+), T_CM_ (central memory T, CD45RA^−^CCR7^+^), T_EM_ (effector memory T, CD45RA^−^CCR7^−^) and T_TD_ (terminally differentiated effector T, CD45RA^+^CCR7^−^). The higher proportion of T_CM_ was found in N15 CAR-T cell group than that in NT-T and N CAR-T cell groups (*P* < 0.001; Fig. 3A-F), while the higher proportion of T_EM_ was found in N CAR-T cell group (*P* < 0.001; Fig. 3A-F).

The subsets of CD4^+^/CD8^+^ (Supplementary Fig. 8A, B), specific activation markers (CD25 and CD69) (Supplementary Fig. 8C-F) and exhaustion markers (PD-1, LAG-3 and TIM-3) (Supplementary Fig. 8G-L) were evaluated in the meanwhile. The findings revealed a significantly increased ratio of CD8/CD4, higher expressions of CD25 and CD69 as well as PD-1 and LAG-3 in N and N15 CAR-T cell groups compared to NT-T cell group (*P* < 0.001), but no statistically significant differences between N and N15 CAR-T cell groups (*P* > 0.05).

The transcriptomes of NT-T, N and N15 CAR-T cells were analyzed (Supplementary Table 4), of which the sample with RNA integrity number (RIN) > 7 was subjected to analyze and to get more than 8G clean data and more than 600 million reads. The sequencing saturation > 75% was subjected to the subsequent analysis. PCA was performed to visualize the clustering and possible outliers of various groups, revealing that N CAR-T cell group exhibited a certain level of clustering with both NT-T and N15 CAR-T cell groups (Supplementary Fig. 9A). This observation indicates that the gene expression characteristics of N CAR-T cell group lie in those between other two groups. With the threshold of log2-fold change > 2.0 and Q < 0.05, both N and N15 CAR-T cell groups exhibited up to thousands of Deferentially Expressed Genes (DEGs) with NT-T cell group, with only a relatively small number of DEGs between N and N15 CAR-T cell groups (Supplementary Fig. 9B). A heatmap of the top 15 DEGs sorted from the small to the large according to *P* values is displayed in Fig. 3G-I. The DEGs related to the function of NKG2D CAR, such as Solute Carrier Family 26 member 11 (SLC26A11), Low Density Lipoprotein Receptor (LDLR), and CX3C Chemokine Receptor1 (CX3CR1), as well as those related to the IL-15/IL-15Rα, such as Filamin A Interacting Protein 1 Like Gene (FILIP1L), Cytosolic Phospholipase A2 (PLA2G4A) and Megakaryocyte-Associated Tyrosine Kinase (MATK) were identified. The GO enrichment analysis revealed that the DEGs in terms of plasma membrane, extracellular region and kinase activity were the most significant in the function of NKG2D CAR (*P* < 0.05; Supplementary Tables 5–7), and the DEGs in terms of extracellular region, nuclear envelope, and nucleotidyltransferase activity were the most significant in the function of IL-15/IL-15Rα (*P* < 0.05; Supplementary Tables 8–10). By the KEGG pathway enrichment analysis, pathways related to cytokine-cytokine receptor interaction, PI3K-Akt signaling pathway, protein digestion and absorption and Jak-STAT signaling pathway, etc. were found to be meaningful targets in future mechanism research (Fig. 3J-L). Besides, the pathways of HALLMARK_E2F_ TARGETS, HALLMARK_MYC_TARGETS and GOCC_PROTEASOME, etc. were screen by GSEA method.

### Characterization of B-LCL cell models

**Fig. 4:**
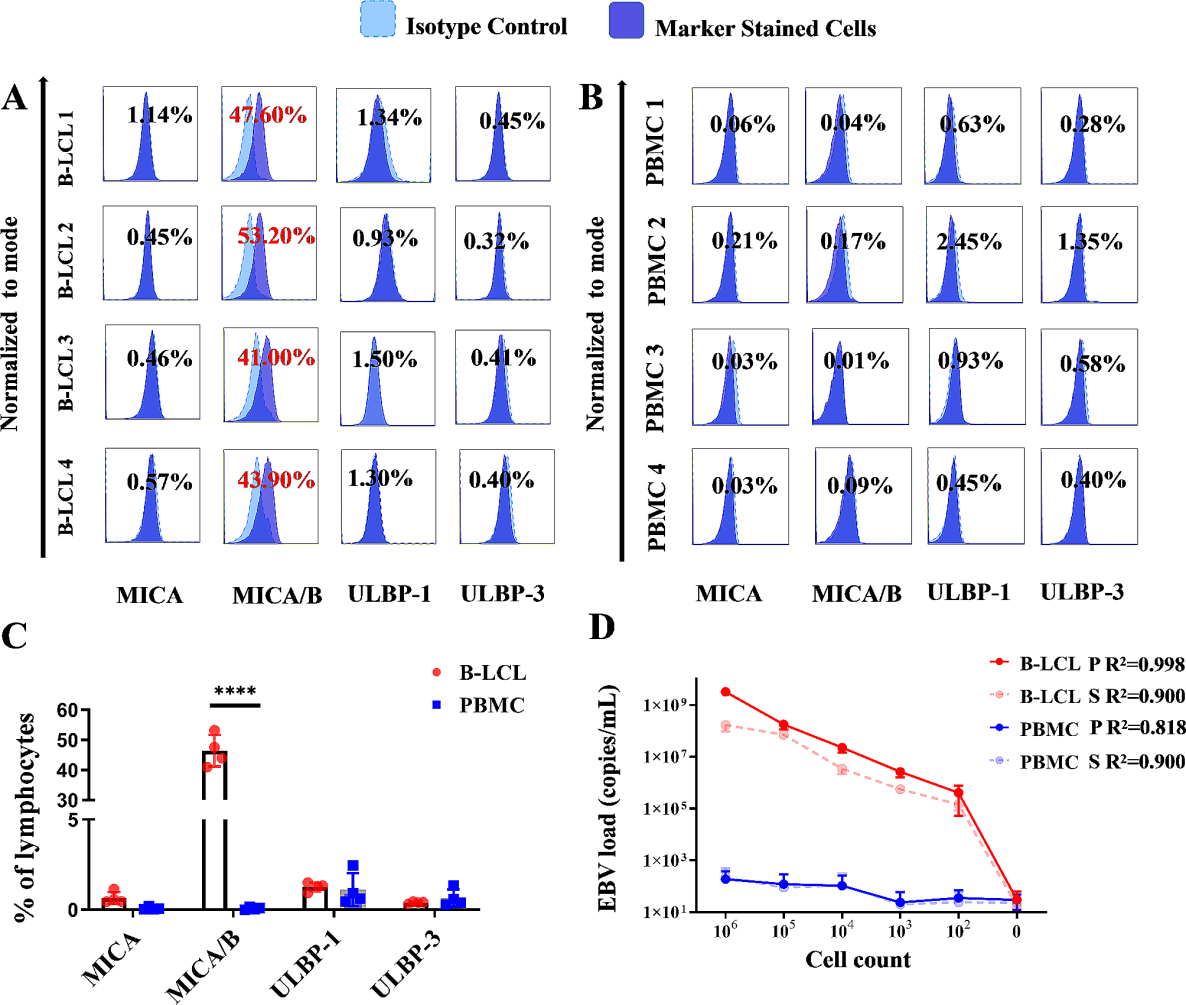
Characterization of the established B-LCL models in comparison with normal PBMCs. (**A**) Flow cytometric histograms of NKG2DLs (MICA, MICA/B, ULBP-1 and ULBP-3) expression on four B-LCLs (derived from 4 individual donors). (**B**) Flow cytometric histograms of NKG2DLs (MICA, MICA/B, ULBP-1 and ULBP-3) expression on four PBMCs (*n* = 4). (**C**) Statistic graph of NKG2DLs (MICA, MICA/B, ULBP-1 and ULBP-3) expression on B-LCLs and PBMCs (*n* = 4). (**D**) EBV DNA load in the cell precipitates (P) and supernatant (S) of B-LCL and PBMCs cultures according to cell counts detected by real-time quantitative PCR. * *P* < 0.05, ** *P* < 0.01, *** *P* < 0.001, **** *P* < 0.0001

Four B-LCL cell lines derived from four individual healthy donors’ PBMCs were detected for MICA, MICA/B, ULBP-1 and ULBP-3 of NKG2DLs on the cell surface by flow cytometry, in which the frequencies of NKG2DL MICA/B on B-LCLs were 46.43 ± 4.56% (Fig. 4A), while significantly lower surface expression of NKG2DLs (0.01–0.17%) was presented on normal PBMCs (*P* < 0.0001; Fig. 4B and C).

The numbers of 1 × 10^6^, 1 × 10^5^, 1 × 10^4^, 1 × 10^3^, 1 × 10^2^ and 0 B-LCLs or PBMCs were seeded in 200 µl RPMI complete medium and then incubated for 8 h. EBV DNA load in B-LCLs and PBMCs were detected by RT-qPCR, of which EBV load was ≥ 1.44 × 10^5^ copies/mL in the cultures of B-LCL lines, positively correlating to cell numbers (precipitates: *R*^*2*^ = 0.998, *P*<0.0001; supernatants: *R*^*2*^ = 0.900, *P*<0.0001; (Fig. 4D). A trace amount of viruses was detected in PBMCs (≤ 227.5 copies/ml), which was a base line of assay and might be related to EBV latent infection of PBMCs (Fig. 4D).

### Killing effect of CAR-T cells on B-LCLs in-vitro

**Fig. 5:**
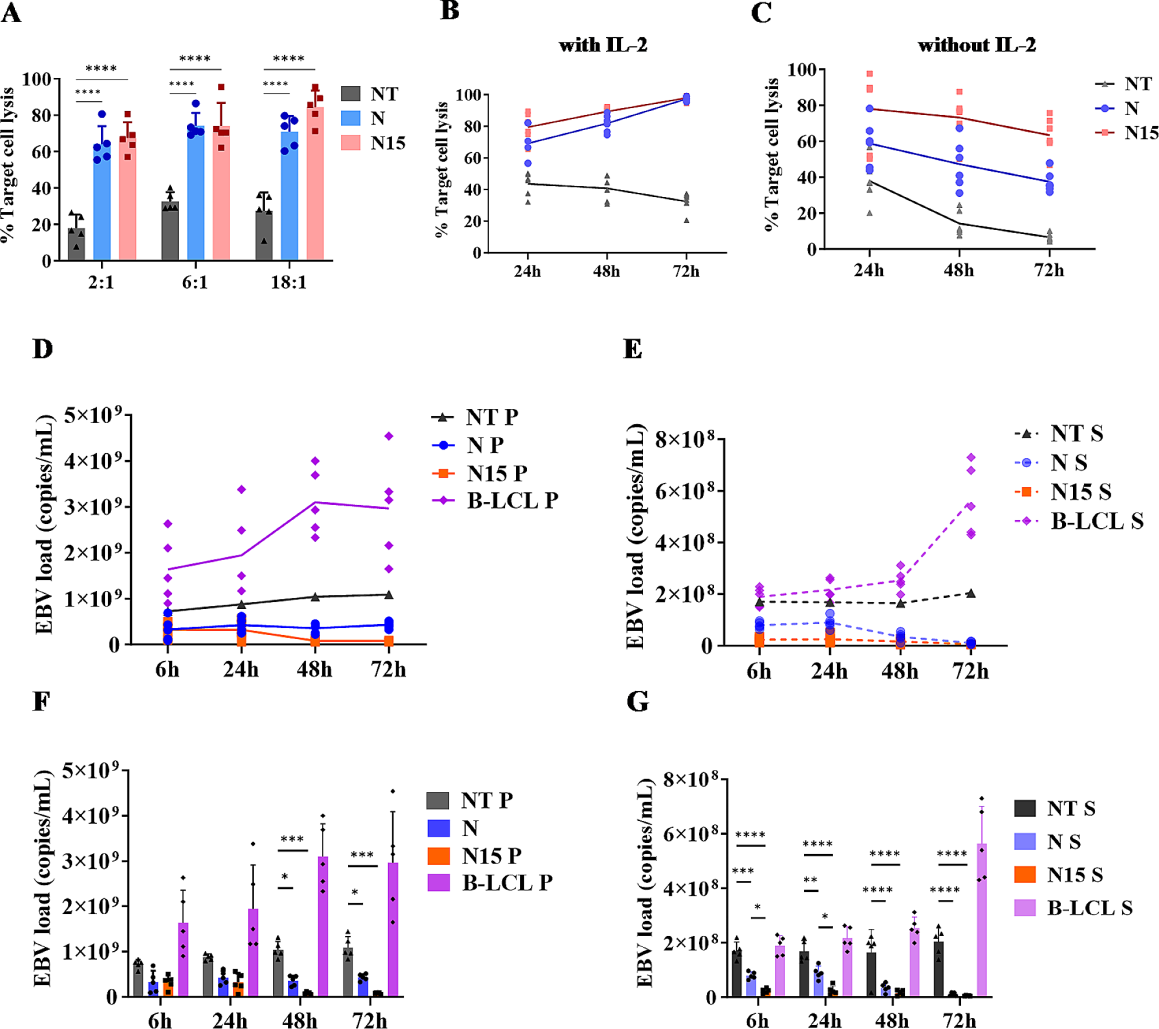
In vitro killing of B-LCLs and inhibiting of EBV replication by three types of T cells. (**A**) Testing for killing effect on B-LCLs in 6 h co-culturing without IL-2 by various types T cells at ratio of 2:1, 6:1 and 18:1, respectively. (**B**,**C**) Lysis of B-LCLs in co-culturing for different time by various type of T cells with/without IL-2 maintenance. (**D**,**E**). Kinetic trend of EBV DNA load in the precipitates (P) and supernatants (S) of co-culturing of B-LBLs and various types of T cells without IL-2 (*n* = 5). (**F**,**G**). Comparison of EBV DNA load in the precipitates (P) and supernatants (S) of co-cultures of B-LBLs between various types of T cells at different time. The data were obtained from 5 independent tests. * *P* < 0.05, ** *P* < 0.01, *** *P* < 0.001, **** *P* < 0.0001

B-LCLs as target cells were measured by the Flow cytometry Absolute Counting Beads (Supplementary Fig. 6). Three types of T cells (5 × 10^5^/500µL) from NT-T, N and N15 CAR-T cell groups were co-cultured with B-LCLs at ratios of 2:1, 6:1 and 18:1 for 6 h without IL-2, and the lysis of B-LCLs was calculated with formula. Both N and N15 CAR-T cell groups exhibited significantly higher killing efficiency compared to NT-T cell control group (*P* < 0.0001, Fig. 5A). The percentage of B-LCLs lysis in NT-T cell group was determined for 17.93 ± 6.69% at 2:1, and slightly increased (32.65 ± 4.50%) at 6:1 and stable (27.49 ± 9.01%) at 18:1 ratios of T cells/B-LCLs, respectively. In N15 CAR-T cell group, the percentage of B-LCLs lysis was from 67.47 ± 7.74% at 2:1 to 84.44 ± 8.17% at 18:1 ratios of N15 CAR-T cells/B-LCLs, significantly higher than that in NT-T cell group (*P* < 0.0001). The B-LCLs lysis rates of N CAR-T cell group were slightly lower than those of N15 CAR-T cell group, but not significantly different from N15 CAR-T cell group (*P* > 0 0.05, Fig. 5A).

When co-culturing of T cells/B-LCLs prolonged to 72 h under IL-2 maintenance, the B-LCLs lysis rates maintained constantly increasing and approaching to 97.37 ± 1.34% or 97.94 ± 1.66% in N and N15 CAR-T cell groups compared to 32.45 ± 5.65% in NT-T cell group (*P* < 0.0001, Fig. 5B); while co-culturing of T cells/B-LCLs from 24 to 72 h without IL-2 maintenance, the B-LCLs lysis rate of N15 CAR-T cell group still exhibited an higher level from 78.01 ± 20.40% to 63.38 ± 8.42% compared to that from 58.78 ± 8.70% to 37.42 ± 2.95 of N CAR-T cell group or from 37.87 ± 11.75% to 6.54 ± 2.13% of NT-T cell group, respectively (*P* < 0.001, Fig. 5C).

Meanwhile, the inhibitory effect of CAR-T cells on EBV DNA replication was measured by RT-qPCR. Under culture condition without IL-2 for 72 h incubation, EBV DNA load with no co-culturing of T cells increased to > 3 × 10^9^ copies/ml in precipitates or > 5 × 10^8^ copies/ml in supernatants of B-LCL controls, respectively (Fig. 5D, E); while with co-culturing of T cells, EBV loads massively decreased in precipitates and supernatants of B-LCL co-cultures with NT-T, N and N15 CAR-T cells (Fig. 5D, E), of which EBV load was the lowest to 5.60 × 10^7^ copies/ml in cell precipitates or 1.10 × 10^7^ in supernatants in N15 CAR-T cell group, significantly lower than that 10^9^ or 10^8^ copies/ml in NT-T cell group (*P* < 0.001, Fig. 5F, G).

### The functionality of NKG2D CAR-T cells

**Fig. 6:**
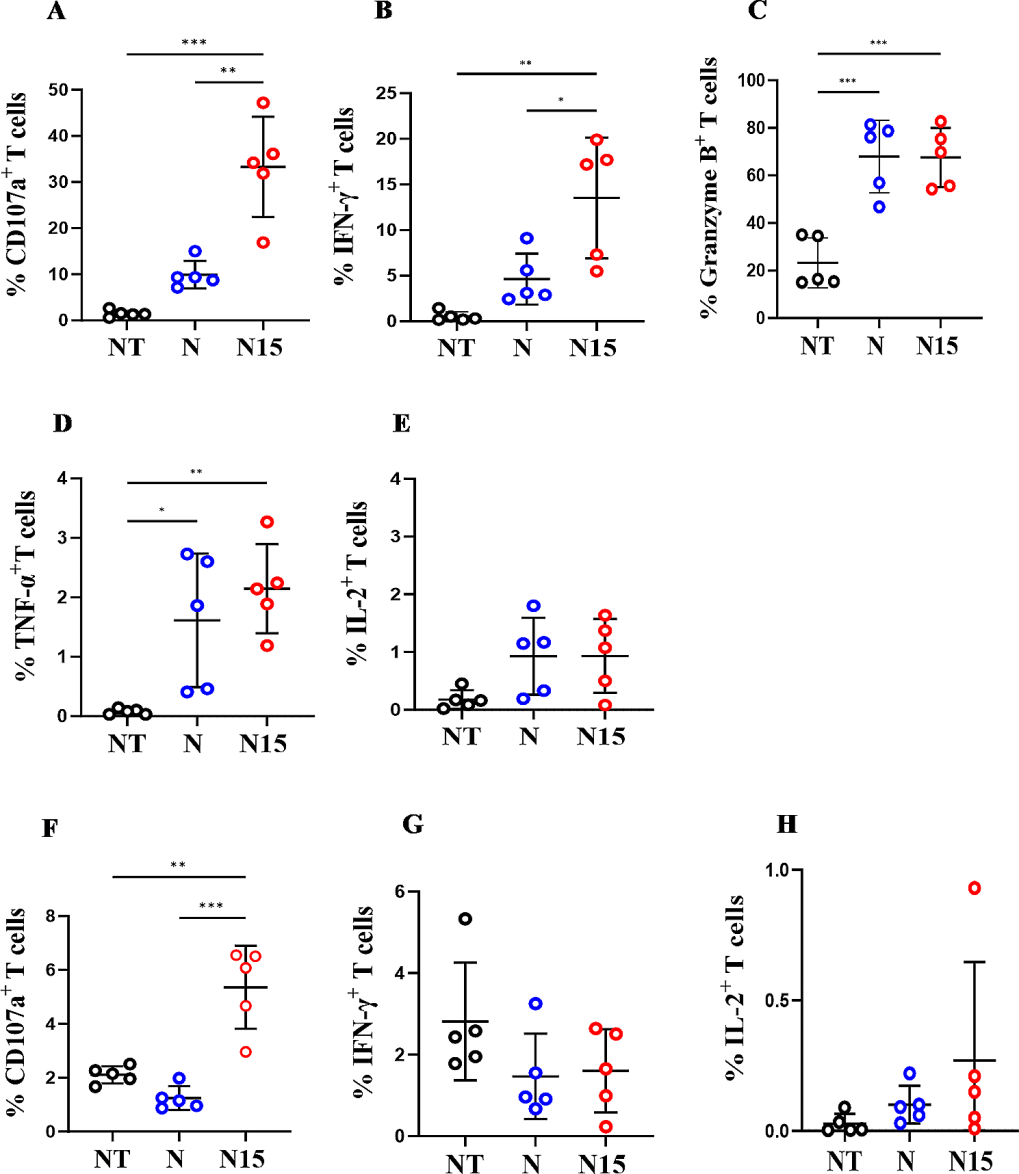
Frequency of cytokine expressing T cells from NT-T, N and N15 CAR-T cell groups in co-culturing with B-LCLs or PBMCs without IL-2 maintenance measured by ICS. (**A**-**E**) In co-culturing with B-LCLs; (**F**-**H**) In co-culturing with PBMCs. The data were presented as the mean of 3 independent tests from 5 individual donors. * *P* < 0.05, ** *P* < 0.01, *** *P* < 0.001, **** *P* < 0.0001

To understand the function mechanism of N15 CAR-T cells targeting B-LCLs, the roles for degranulation and lysis of B-LCLs were evaluated by ICS assays. Significantly higher CD107a and IFN-γ expressions were found in N15 CAR-T cell group compared to N CAR-T and NT-T cell groups (*P* < 0.01; Fig. 6A and B), while equally higher TNF-α and Granzyme B expressions were in N15 and N CAR-T cell groups compared to NT-T cell group when co-cultured with B-LCLs (*P* < 0.01; Fig. 6C and D). No notable difference was observed in IL-2 expression level among the three groups (*P* > 0.05; Fig. 6E). In addition, the cytokine expressions were not significantly elevated excepting for relatively higher CD107a in N15 CAR-T cell group when these three types of T cells co-cultured with normal PBMCs (no NKG2DLs) instead of B-LCLs (*P* > 0.05; Fig. 6F-H), indicating the targeting specificity of NKG2D CAR-T cells.

Additionally, the secretion cytokines were dynamically assessed in 6–72 h co-cultures of B-LCLs with three types of T cells from TN-T, N and N15 CAR-T cell groups by ELISA (Supplementary Fig. 10). A substantial amount of IL-15/IL-15Rα (> 1000pg/ml) was consistently detected only in N15 CAR-T cell group but not in NT-T and N CAR-T cell groups (*P* < 0.0001; Supplementary Fig. 10A). The level of IL-2, IL-10, IFN-γ and GM-CSF secretions was observed relatively higher from N15 CAR-T cell group than those from N CAR-T cell group (*P* < 0.01; Supplementary Fig. 10B-E), of which both groups were significantly higher than those from NT-T cell group (*P* < 0.001). A transient secretion of TNF-α was found in 6 h co-cultures from N15 CAR-T cell group, significantly higher than that from N CAR-T and NT-T cell groups (*P* < 0.001; Supplementary Fig. 10F). Notably, the secretion level of IL-6 from N15 CAR-T cell group was relatively lower than that from N CAR-T group (*P* < 0.05), of which both reached to the top high in 24 h co-cultures and then declined to the low level in 72 h co-cultures, but still higher than that from NT-T cell group (*P* < 0.01; Supplementary Fig. 10G). These results suggested that N15 CAR-T cells produced the higher levels of various cytokines, which played the important roles attributing to the cytotoxicity effect on B-LCLs and maintained in the safety state.

### Therapeutic efficacy of NKG2D CAR-T cells on EBV-PTLD mouse models

**Fig. 7:**
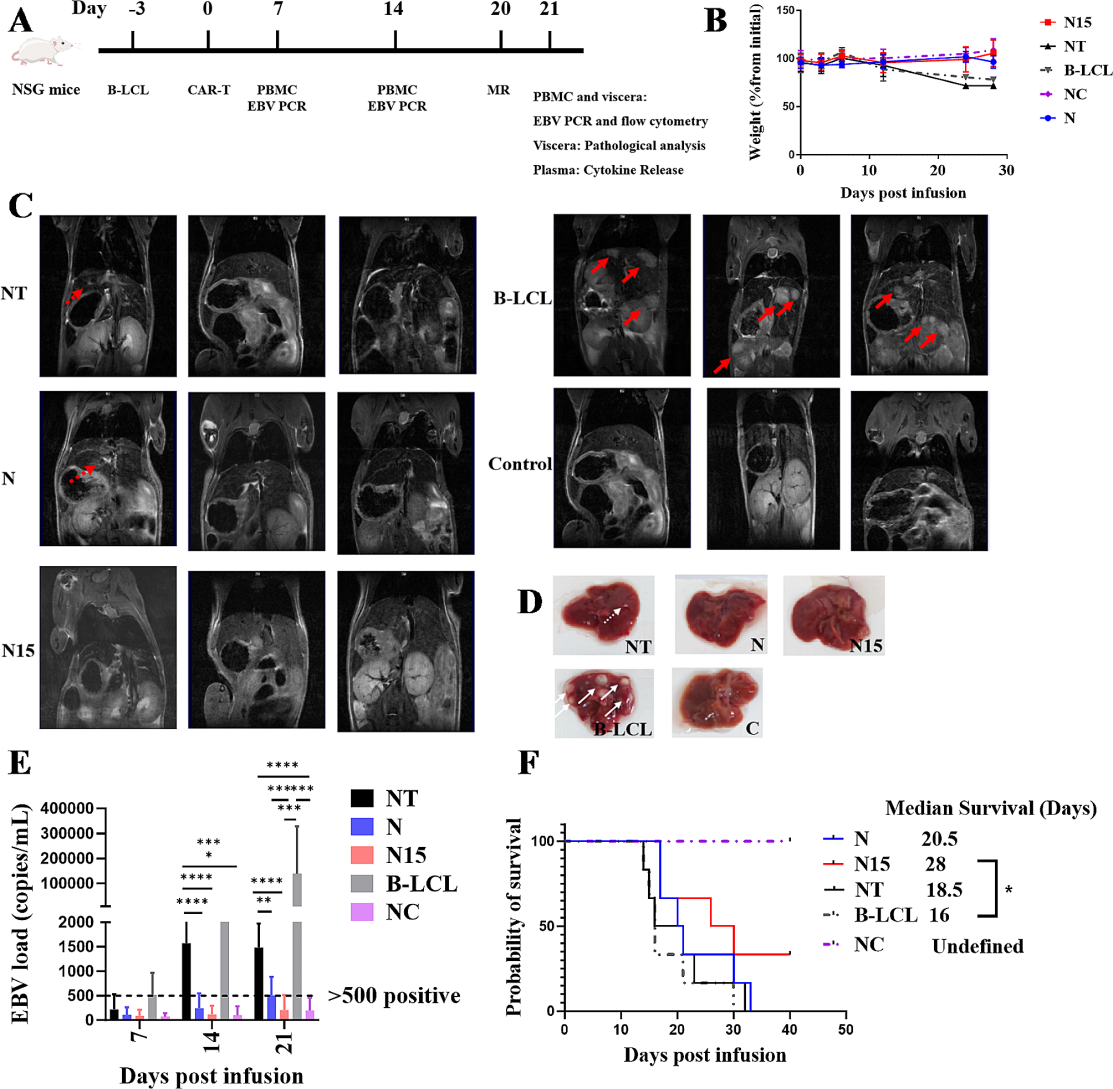
Examination for clinical outcomes of EBV-PTID mouse models treated by three types of T cells. (**A**) Schematic schedule of treatment and examination for B-LCLs xenografted NSG mice (EBV-PTID mouse models). (**B**) Weight curve (*n* = 6). (**C**) MR imaging of treated and control mice (*n* = 3). The red solid line arrows showed the tumor signals; red dashed arrows showed the suspected tumor signals. (**D**) Pathological observation for representative livers of treated and control mice. The white solid line arrows showed the tumor signals; white dashed arrows showed the suspected tumor signals. **(E)** EBV DNA load in caudal venous blood of treated and control mice detected by qPCR (*n* = 6). (**F**) Kaplan–Meier survival curve (*n* = 6). * *P* < 0.05, ** *P* < 0.01, *** *P* < 0.001, **** *P* < 0.0001

To evaluate the therapeutic efficacy of CAR-T cells in vivo, B-LCLs xenografted NSG mice as EBV-PTLD models were classified to four groups and then treated by PBS (B-LCL group), NT-T, N and N15 CAR-T cells, respectively (Fig. 7A). By examining the body weight, NT-T cell and B-LCL groups appeared to the most reduction, while N15 CAR-T cell and NC groups were slightly increased, which were significantly higher than NT-T cell and B-LCL groups (*P* < 0.001; Fig. 7B).

The tumor progression in mouse models was assessed by micro-MRI imaging, and EBV DNA load was detected from tail vein blood. Generally, the tumor tissues exhibited intermediate to high signal intensity on T2-weighted imaging (T2WI) and appeared to white or white-gray (Fig. 7C). In B-LCL group with PBS treatment, the numbers of tumor foci signals were observed in all three mice, distributed in various locations such as the liver, intestine, and kidney. These tumors presented as either masses or adhering to surrounding tissues, which were consistent with the gross pathology (Fig. 7D). Among three groups of NT-T, N or N15 CAR-T cell treated B-LCL mice, a mouse liver exhibited scattered area of hyper-intensity in NT group (Fig. 7D), that was confirmed to be tumor tissue upon dissection; a suggestive sign of tumor was displayed in a mouse of N CAR-T cell treated group, but no actual tumor was found after dissection; no signal of tumor foci was observed in N15 CAR-T cell and naïve mouse control (NC) groups. By anatomical pathological observation of liver organs from these five groups of mice, obviously more and clear tumor nodules were found in B-LCL mice, fewer and lighter nodules were observed in NT-T and N CAR-T cell treated mice, while no tumor nodule was seen in both N15 CAR-T cell treated and NC mice.

By quantifying EBV DNA load in blood samples from EBV-PTLD mouse models during 7 to 21 days post infusion and treatment, both N and N15 CAR-T cell groups had low level of EBV viremia at detection base line (500 copies/ml), of which N CAR-T cell group was from 108 ± 140 copies/mL (Day 7) to 506 ± 346 copies/mL (Day 21), while N15 CAR-T cell group was from 88 ± 113 copies/mL (Day 7) to 205 ± 283 copies/mL (Day 21) similar to NC group. In the control groups, EBV viremia increased to 1500 copies/ml in NT-T cell group and > 1 × 10^5^ copies/ ml in B-LCL group at 21 days post treatment, respectively, which were significantly higher than those in N and N15 CAR-T cell groups (*P* < 0.0001). Naïve NGS mice were treated by PBS as non-tumor bearing normal animal control (NC) (Fig. 7E).

Finally, among five groups of mice, the ranking of mean survival time was NC (> 50 days) > N15 (40 days) > N (33 days) > NT (32 days) > B-LCL (30 days) (Fig. 7F). Notably, N15 CAR-T cell group demonstrated significantly prolonged survival time compared to B-LCL group (*P* = 0.0478; Fig. 7F). However, most mice in experimental groups died 40 days after treatment probably because of immunodeficiency in NSG mice.

In an overall, IL-15/IL-15Rα-NKG2D CAR-T cells (N15 CAR-T cell group) had the most satisfactory efficacy for inhibiting B-LCL tumor progression and EBV replication in mouse models.

### Functional analysis of NKG2D CAR-T cells in B-LCLs xenografted mice

**Fig. 8:**
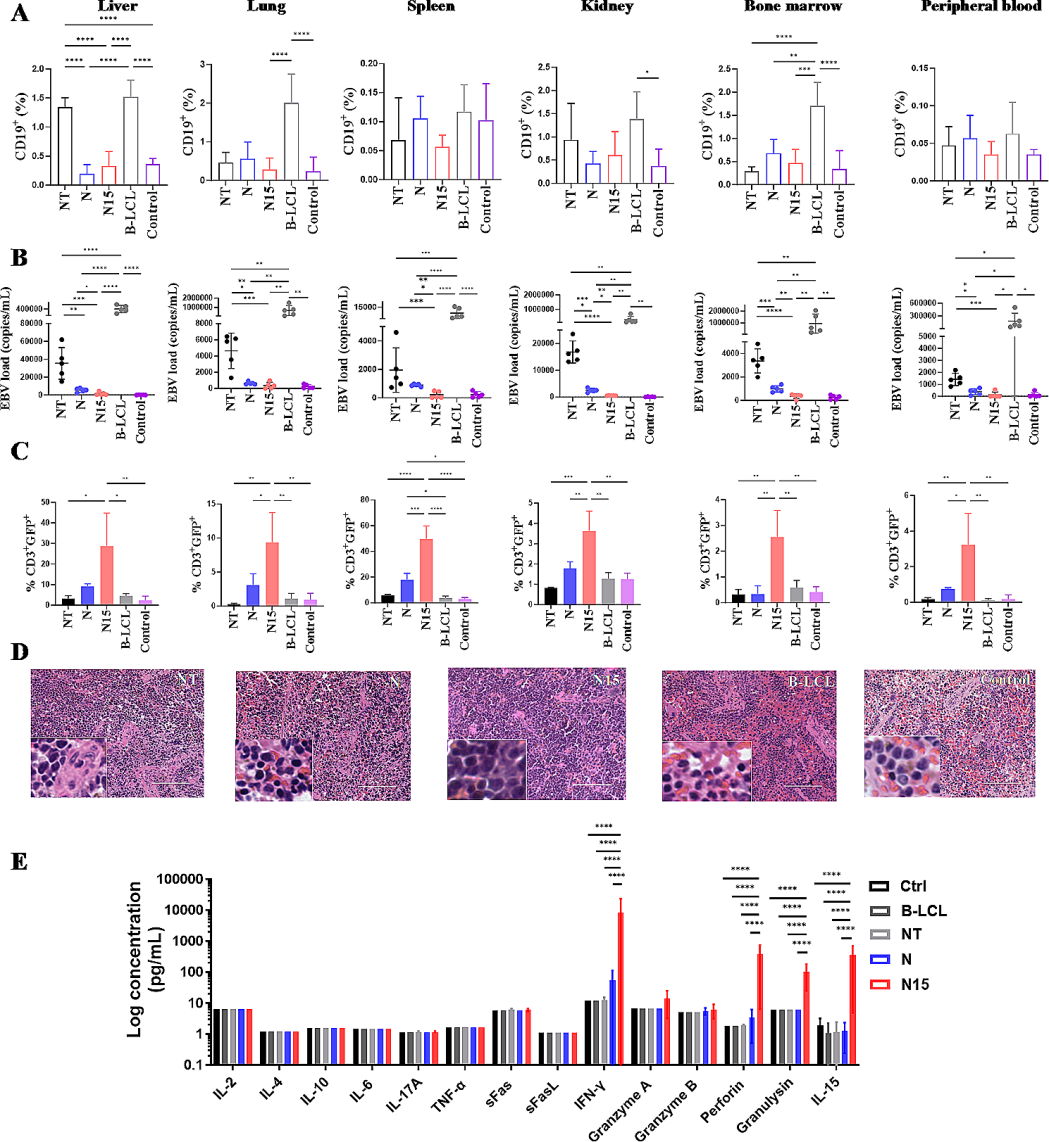
Evaluation for effects of three types of T cells for treating EBV-PTID mouse models. (**A**) B-LCL tumor burden in the liver, lung, spleen, kidney, bone marrow and peripheral blood of mice detected by flow cytometry (*n* = 6). (**B**) EBV DNA load in the liver, lung, spleen, kidney, bone marrow and peripheral blood of mice detected by qPCR (*n* = 6). (**C**) T cells resided in the liver, lung, spleen, kidney, bone marrow and peripheral blood evaluated by flow cytometry (*n* = 6). (**D**) H&E staining of spleen tissue sections. Scale = 400 μm. The bottom left region is of 200-fold magnification. (**E**) Measurement of cytokines in blood of mice by Multiplex Human Cytokines Analysis (*n* = 6). Significantly high level of IL-15, IFN-γ, Perforin and Granulysin were detected in N15 CAR-T cell group. * *P* < 0.05, ** *P* < 0.01, *** *P* < 0.001, **** *P* < 0.0001

Six NSG mice from each group were sacrificed on Day 21 post treatment, the various tissues and peripheral blood samples were collected to evaluate the B-LCL tumor burden, EBV DNA load, T cell distribution and cytokine release in vivo. The tumor burden of CD19^+^ B-LCLs was measured by flow cytometry in liver, lung spleen, kidney, bone marrow tissues and blood from five groups (Gating strategy: Supplementary Fig. 11A). The highest proportion (> 1%) of B-LCLs was detected in all tissues from B-LCL group, while the lowest proportion (< 0.5%) of B-LCLs was found in most tissues from N15 CAR-T cell group similar to NC group, and the significant difference was found in liver, lung, kidney and bone marrow tissues between N15 CAR-T cell and B-LCL groups (*P* < 0.05; Fig. 8A).

EBV DNA load was also measured in various tissues and blood samples (Fig. 8G-L), in which EBV load was lowest or undetectable from N15 CAR-T cell and NC groups compared with NT-T cell group (< 500 copies/ml vs. 1415–16827 copies/ml; *P* < 0.001) and B-LCL group (< 500 copies/ml vs. 9799-951698 copies/ml; *P* < 0.001), suggesting N15 CAR-T cells had strongly antiviral effect. N CAR-T cell group had higher viral load than N15 CAR-T cell group (*P* < 0.05), but significantly lower than NT-T cell group (*P* < 0.001). Notably, N15 CAR-T cell group had low viremia (116 ± 220 copies/ml in blood; Fig. 8L), which approximately were 1500 times lower than B-LCL group (177220 ± 168131 copies/ml in blood). EBV DNA was undetectable (< 500 copies/ml) in spleen and bone marrow tissues from N15 CAR-T cell treated group (Fig. 8B).

The distribution of CD3^+^ T or EGFP^+^ CAR-T cells was examined quantitatively by flow cytometry in the various tissues and peripheral blood from all treated animal groups (Gating strategy: Supplementary Fig. 11B and C). The proportion of CAR-T cells was significantly higher in all tissue samples from N15 CAR-T cell group compared to N CAR-T and NT-T cell groups (*P* < 0.05; Fig. 8C), while no statistical difference was observed between N CAR-T and NT-T cell groups (*P* > 0.05), suggesting that the higher proportion of N15 CAR-T cells might be associated with effect of IL-15/IL-15Rα activity on the cell viability and proliferation. A notably high proportion of CAR-T cells was observed up to 60% in spleen tissue of N15 CAR-T cell group, and a large amount of lymphocyte infiltration were found in spleen tissue by H&E staining (Fig. 8D), which might attribute to the undetectable EBV load in spleen (Fig. 8C).

The cytokine release was evaluated in vivo from five groups of treated EBV-PTLD mouse models on Day 21 by Multiplex Human Cytokines Analysis (Fig. 8E). IFN-γ, Perforin, Granulysin and IL-15 were detected higher in the plasmas of N15 CAR-T cell group than those of N CAR-T and NT-T cell groups, showing the effect of IL-15/IL-15Rα on NKG2D CAR-T cell activity against B-LCLs tumorigenesis and EBV replication.

## Discussions

This study provides novel evidence that NKG2D CAR-T exerts anti-tumor and anti-viral activities in treating EBV-PTLD, and IL-15/IL-15Rα improves the therapeutic and safety effects by increasing the proportion of T_CM_, promoting T cell homing in spleen and bone marrow and multiple synergistical cytokine regulation.

NKG2D has been employed in CAR-T therapy for various hematologic malignancies, such as acute myeloid leukemia (AML) [49] and MM [27]) as well as solid tumors (including colorectal cancer [50] and triple-negative breast cancer [26]. However, research on NKG2D CAR-T therapy for EBV-PTLD was limited. CAR-T targeting EBV antigens and B cell markers were explored, showing certain efficacy in treating EBV-PTLD in clinical and preclinical researches [18–20], but confronted with the limitation of antigen escape and B-cell lineage immune suppression, respectively. In this study, we demonstrated that NKG2D CAR-T exhibited potent cytotoxic effects against B-LCL cells along with specific cytokine secretion. As an alternative therapeutic approach for EBV-PTLD, NKG2D CAR-T combining with other types of CAR therapy could prevent immune escape from antigen loss and immune suppression and provide additional treatment options for patients.

Recently, the development of NKX101, a generic NKG2DL-targeted CAR-NK therapy by Nkarta, is being suspended due to its rapid CAR-cell exhaustion and transient curative effects failing to prevent relapse. We found that IL-15/IL-15Rα benefit the persistence of CAR-T cells *in vivo.* Previous studies have demonstrated that activation of JAK-STAT pathway is responsible for promoting NK/T cell proliferation by IL-15 through binding with L-2/IL-15Rβγ [51, 52]. Here, our transcriptome sequencing data confirmed that JAK-STAT, PI3K-Akt and protein digestion and absorption signaling pathway enrichment in comparison between N and N15 groups, of which molecular mechanisms remain to be established.

Besides, IL-15/IL-15Rα signaling promoted the expansion of less differentiated T_CM_. An increasing amount of evidence supported that sustained remission and improved outcomes in patients with tumors were associated with an elevated frequency of T cells possessed memory-like characteristics [53–55]. The more differentiated T_CM_ exhibits lymphoid homing property and possesses stronger proliferative capacity, while T_EM_ tends to produce diverse effector cytokines, and the viability, persistence, and anti-tumor ability are superior for T_CM_ when compared with T_EM_ counterparts [56–61]. In our study, IL-15/IL-15Rα induced a greater proportion of T_CM_ (CD45RA^−^ CCR7^+^) cell differentiation, suggesting a superior therapeutic effects on IL-15/IL-15Rα NKG2D CAR-T cells. Moreover, IL-15/IL-15Rα promoted the efficacy and duration of therapeutic effects in vivo probably by promoting NKG2D CAR T cell homing in spleen and bone marrow. The rich accumulation of injected CAR cells in the immune tissue and organ niches was proved to be related to the efficacy and duration of CAR therapy [62, 63].

Importantly, we observed that superior impact of NKG2D CAR and IL-15/IL-15Rα on suppressing EBV replication and regulating viral dissemination, both of which theoretically exert cytotoxic effects on virus-infected cells. During the reactivation phase of lytic replication, EBV generates a substantial number of infectious virions that infect new host cells to maintain the EBV reservoir in the body and facilitate continuous viral dissemination. Previous studies have developed CAR-T containing single-chain variable fragments (scFv) targeting EBV-infected host cell glycoprotein gp350 [15] or lytic phase protein LMP-1 [17] demonstrating their cytotoxic effects on 293-T cells expressing these target proteins. Co-culturing with EBV^+^ B-958 cells effectively eliminates target cells and induces the release of immune-specific cytokines such as IFN-γ. In this study, we focused on NKG2D as the target molecule and compared it with scFv-based CAR-Ts using a human sequence-based NKG2D CAR structure that does not elicit xenogeneic immune responses. In vivo experiments confirmed that the NKG2D CAR structure significantly reduced EBV DNA load in peripheral blood and tissues. Importantly, integrating IL-15-IL-15Ra further enhanced this effect, potentially due to increasing of T_CM_ and IFN-γ, Perforin, and Granulysin production. Our findings suggest that NKG2D CAR-T therapy may effectively control EBV dissemination through rapid elimination of infected host cells and secretion of cytokines such as IFN-γ, Perforin, and Granulysin. Further investigation is warranted to elucidate the microscopic process and mechanism underlying the interaction between CAR-T and viral infection.

The B-LCL cell line is a commonly used model in immunotherapy experiments for PTLD [64, 65]. NKG2D ligands MICA/B were found approximately 40–50% on the surface of B-LCLs. Despite only an half of target cells expressing NKG2DLs, the CAR-T therapy could still exert bystander effects by eliminating antigen-negative tumor cells through various cytokine secretions. This theory was also demonstrated by effective NKG2D CAR-T treatment for acute myeloid leukemia (AML) patients [66]. In theory, the significantly up-regulated NKG2DL expressions on EBV-infected cells enable the cells to be recognized, bound, and directly killed by NKG2D CAR-T or IL-15/IL-15Rα-NKG2D CAR-T cells. In our study, we demonstrated that IL-15/IL-15Rα-NKG2D CAR-T cells massively inhibited EBV replication in vitro and in vivo, of which EBV load was reduced more than 1000 times in various tissue and blood compared with control groups.

In this study, the EBV-PTLD model was established by xenografting human B-LCLs into NSG mice, and a high B-LCL tumor engraftment rate with high level of EBV DNA load was demonstrated. However, it should be noted that these mice lacked an entire human immune system, limiting the measurement of the cascade reactions induced by cytokine release syndrome (CRS), neurotoxicity, and autoimmune response, which were common reactions following CAR-T cell treatment causing by the host’s own immune system [67]. Therefore, further evaluating the efficacy and safety profiles of IL-15/IL-15Rα-NKG2D CAR-T cells in humanized mice model is warranted. In addition, HLA typing of T cells and target cells is essential for the future experiment.

The proposal of this study was based on the theoretical immune cytotoxicity effect of NKG2D CAR and IL-15-IL-15Ra on EBV-infected cells. For other virus-related tumors, such as other EBV-related lymphoma and nasopharyngeal carcinoma, human papillomavirus, hepatitis virus, and even human immunodeficiency virus-induced tumor, as well as acute viral infectious diseases, including infectious mononucleosis induced by EBV, cytomegalovirus pneumonia, and COVID-19, this treatment may play a role in depending on the expression of NKG2DLs on target cells.

## Conclusions

The co-expressed IL-15/IL-15Rα complex could prolong the proliferation and survival of NKG2D CAR-T cells, resulting in superior anti-tumor and antiviral effects in EBV-PTLD models. This study presents an effective and safety approach utilizing IL-15/IL-15Rα-NKG2D CAR-T cells for the treatment of EBV-PTLD.

## Electronic supplementary material

Below is the link to the electronic supplementary material.

Supplementary Material 1

## Data Availability

No datasets were generated or analysed during the current study.

## Abbreviations

AML: Acute myeloid leukemia
B-LCL: B-lymphoblastoid cell line
CAR-T cell: Chimeric antigen receptor T cells
CRS: Cytokine release syndrome
EBV: Epstein-Barr virus
EBV-CTL: EBV-specific T cells
EBV-PTLD: EBV related post-transplant lymphoproliferative disorder
HBV: Hepatitis B virus
HCV: Hepatitis C virus
HHV4: Human herpes virus 4
HPV: Human papillomavirus
HSCT: Hematopoietic stem cell transplantation
Micro-MRI: Micro-magnetic resonance imaging
NKG2D: Natural killer group 2 member D
NKG2DLs: NKG2D ligands
SOT: Solid organ transplantation
