# Correction: Efficacy of NKG2D CAR-T cells with IL-15/IL-15Rα signaling for treating Epstein-Barr virus-associated lymphoproliferative disorder

**DOI:** 10.1186/s40164-024-00559-7

**Published:** 2024-09-12

**Authors:** Qiusui Mai, Bailin He, Shikai Deng, Qing Zeng, Yanwen Xu, Cong Wang, Yunyi Pang, Sheng Zhang, Jinfeng Li, Jinfeng Zeng, Liqin Huang, Yongshui Fu, Chengyao Li, Tingting Li, Xiaojun Xu, Ling Zhang

**Affiliations:** 1 Department of Blood Transfusion, The Seventh Affiliated Hospital, Sun Yat-Sen University, Shenzhen, 518107 China; 2 Department of Transfusion Medicine, School of Laboratory Medicine and Biotechnology, Southern Medical University, Guangzhou, 510515 China; 3 Department of Hematology, Nanfang Hospital, Southern Medical University, Guangzhou, 510515 China; 4 Department of Obstetrics, He Xian Memorial Affiliated Hospital of Southern Medical University, Guangzhou, 511402 China; 5 Guangzhou Bai Rui Kang (BRK) Biological Science and Technology Limited Company, Guangzhou, 510555 China; 6 Department of Obstetrics, Nanfang Hospital, Southern Medical University, Guangzhou, 510515 China; 7 Shenzhen Bao’an District Central Blood Station, Shenzhen, 518101 China; 8 Shenzhen Blood Center, Shenzhen, 518035 China; 9 Guangzhou Blood Center, Guangzhou, 510095 China

**Correction: Experimental Hematology & Oncology (2024) 13:85** 10.1186/s40164-024-00553-z

In this article [1], authors Qiusui Mai, Bailin He and Shikai Deng should have been denoted as equally contributing authors. The equal contribution information was inadvertently omitted during the production process.

The original article has been corrected.

## References

[1] Mai Q, He B, Deng S, Zeng Q, Xu Y, Wang C, Pang Y, Zhang S, Li J, Zeng J, Huang L, Fu Y, Li C, Li T, Xu X, Zhang L. Efficacy of NKG2D CAR-T cells with IL-15/IL-15Rα signaling for treating Epstein-Barr virus-associated lymphoproliferative disorder. Exp Hematol Oncol. 2024;13:85. 10.1186/s40164-024-00553-z. 39160631 10.1186/s40164-024-00553-z PMC11334566

