# Supplementary material for: Efficacy of NKG2D CAR-T cells with IL-15/IL-15Rα signaling for treating Epstein-Barr virus-associated lymphoproliferative disorder
Source: Exp Hematol Oncol. 2024 Aug 19;13:85. doi: 10.1186/s40164-024-00553-z (PMC11334566; doi:10.1186/s40164-024-00553-z)
Supplement: Supplementary file 1 — Supplementary Material 1 [file 40164_2024_553_MOESM1_ESM.docx]

# Supplementary Materials

**Efficacy of NKG2D CAR-T cells with IL-15/IL-15Rα signaling for treating Epstein-Barr virus-associated lymphoproliferative disorder**

# Supplementary Methods

**PBMC isolation**

Twenty milliliters of ficoll (Tianjin Haoyang Biological Manufacture Co., Ltd, China) were added into a 50ml-centrifuge tube followed by an equal volume of “Buffy coat” taken out from the blood bag. Went through the procedure of horizontally centrifugation at 1000g×30 min, cells of lymphocyte layer were aspirated and washed with 50ml PBS at 400g×30 min for twice. Then the cells were re-suspended in 50ml PBS. Total cell numbers were calculated by a counter using trypan blue staining. A total of 3×10^8^ - 8×10^8^ PBMCs could be harvested from an individual Buffy coat.

**B-LCL line construction**

Firstly, viral supernatant was prepared with EBV infected B95-8 cell line. B95-8 cells were cultured in RPMI 1640 medium supplemented with 10% heat-inactivated fetal bovine serum (FBS; Gibco), 100 units/ml penicillin, and 100µg/ml streptomycin (Invitrogen) at 37°C in a humidified 5% CO2 incubator for 2 to 3 passages, then continually cultured for 7 days in RPMI 1640 medium supplemented with 2% FBS and 1% penicillin-streptomycin. The cells were harvested and repeatedly frozen and thawed 3 times at -80 °C. Finally, the cell lysate was centrifuged and filtered with a 0.45 μm filter. Secondly, PBMCs were infected with the viral supernatant. Twenty millions of PBMCs were suspended in culture medium containing 4ml viral supernatant and 6ml RPMI 1640 complete medium supplemented with 2μg/ml cyclosporine A, and cultured at 37°C in a humidified 5% CO2 incubator for 4-6 days. Half volume of culture medium was changed every 4 to 5 days. After stable proliferation, the B-LCL cells were harvested and identified for biological marker and EBV load by flow cytometry (CD 19^+^) and qPCR.

**Real-time quantitative PCR (qPCR) to detect EBV load**

EBV load was measured by qPCR according to the protocol of EBV nucleic acid quantitative detection kit (Beijing Gene Technology Co., Ltd., China). Briefly, cell precipitate, supernatant (50μL), blood (50μL) or grinding fluid of different tissues (relatively equal volume) were collected and went through the procedure of lysing, nucleic acid precipitation and being washed to dry and re-dissolved to extract the nucleic acid. The concentration and purity of isolated DNA samples were determined using Nanophotometer (IMPLEN Nanophotometer, Munich, Germany) before storing at -80°C. PCR amplification was carried out on the AriaMx Real-Time PCR System (Agilent Technologies). In each PCR reaction, the reaction mixture consists of 2 μl DNA (4 quantification standards, negative quality controls, and samples in duplicate), 11.5μl PCR reaction solution (containing forward primer, reverse primer, probe master mix and PCR-graded water), 10 μl PCR enhancer and specific Universal Library probe. The reaction is initiated by pre-incubation at a temperature of 95°C for a duration of 10 minutes. Forty cycles of amplification are performed through DNA denaturation at a temperature of 95°C for a duration of 10 seconds followed by annealing and elongation at a temperature of 60°C for a duration of 30 seconds. Following the completion of the reaction, the credibility of the experiments is validated by the negative quality controls with no S-type amplification curve and 4 quantification standards with S-type amplification curves in HEX channel. Standard curves are generated plotting threshold cycle (Ct) values of known concentration of each quantification standard with |R| (|r|)≥0.99 or R²(r²)≥0.98. The concentration of each sample is calculated according to the standard curve. The lowest detectable limit for the kit is 1×10^3^ copies/ml.

Primer sequence (5’ to 3'):

B-globin Forward: GGCCCTTTTGCTAATCATGT

Reverse: CACACAGACCAGCACGTTG

EBNA1 Forward: GAGAAGGCCCAAGCACTG

Reverse: CTCCTTGACCACGATGCTTT

**Construction and identification of pSFG-****NKG2D CAR and pSFG-IL-15/IL-15Rα-NKG2D CAR plasmids**

The gene fragments of NKG2D CAR and IL-15/IL-15Rα-NKG2D CAR were synthesized by Huada Gene Co., Ltd. (Shenzhen, China) and linked to pMV vectors to construct pMV-NKG2D CAR and pMV-IL-15/IL-15Rα-NKG2D CAR. The NKG2D CAR and IL-15/IL-15Rα-NKG2D CAR genes were inserted into the retroviral vector plasmid pSFG.eGFP (MiaoLingBio, Wuhan, Hubei, China) to get pSFG-NKG2D CAR and pSFG-IL-15/IL-15Rα-NKG2D CAR through molecular cloning techniques. The processes included extracting template plasmid and vector, PCR amplification of the target fragments of NKG2D CAR and IL-15/IL-15Rα-NKG2D CAR, linearizing the pSFG vector by Ncol I and Mlu digestion, gel recovery and ligation of the target fragment and vector, transforming to *Escherichia coli* DH5α, identifying positive clones through PCR, extracting recombinant plasmids and finally identification by endonuclease digestion and Sanger sequencing.

Primer sequences (5’ to 3'):

NKG2D CAR F：CATGCCATGGCCCTGCCCGTGACAGCTCTG Ncol I

NKG2D CAR R：CGACGCGTTTATCTAGGAGGCAGGGCCTGC Mlu I

IL-15/IL-15Rα-NKG2D CAR-T F：CATGCCATGGCCCTGCCCGTGACAGCTCTG Ncol I

IL-15/IL-15Rα-NKG2D CAR-T R：CGACGCGTTTAATGGTGATGGTGATGAT Mlu I

**NKG2D CAR and IL-15/IL-15Rα-NKG2D CAR expression detected by Western blot analysis**

1.5×10^6^ per well of 293T were seeded in a 6-welll-plate and transfected with pSFG-NKG2D CAR, pSFG-IL-15/IL-15Rα-NKG2D CAR or pSFG empty plasmid via Lipofectamine 3000 Transfection Kit (Invitrogen). After transfection of 72h, precipitate and supernatant of 293T cell cultures of each group were harvested respectively and prepared with RIPA lysis buffer to get protein lysates. The proteins were separated by 12% SDS-PAGE electrophoresis, and then transferred to 0.45 μm PVDF membrane with wet transfer method under 100 V constant pressure for 60 min. The membrane was blocked with 5% milk for 1h and incubated with primary antibodies overnight at 4℃, and secondary antibodies for 2h at room temperature before color development using an ECL chemiluminescence chromogenic kit and imaging ChemiDoc MP chemiluminescence imaging system.

Transfection reaction system:

Lipofectamine 3000 mix：200 μl Opti-MEM+6 μl Lipofectamine 3000 Regent.

DNA Master Mix：200 μl Opti-MEM +2 μg pSFG vector plasmid+6 μl P3000 Regent.

Primary antibodies used: Rabbit Anti-Human CD247 for CD3ζ on NKG2D CAR; Mouse His-tag antibody for His-tag on IL-15/IL-15Rα; Rabbit Anti-Human IL-15 for IL-15 on IL-15/IL-15Rα.

**Flow cytometry to detect NKG2D CAR**

On Day 3, 8, 13, and 18 post transduction, 0.5 million of NT-T, N and N15 CAR-T cell groups were collected and washed with 1ml PBS once by centrifugation at 250g for 5min at 4℃. Then cells were re-suspended in 100μl staining buffer (containing 2% FBS in 1×PBS) and incubated with 2μl/test of anti-CD3 PerCP Cy5.5 and anti-human CD314 APC antibodies at 2-8℃. Finally, the cells were washed with 1ml PBS and re-suspended in 200μl PBS for detection by BD LSRFortessa. Data were analyzed with FlowJo v10.

**ELISA to detect IL-15/IL-15Rα**

On Day 8 post transduction, 0.5 million of T cells were extracted from NT, N, and N15 groups (n=5). The cells were suspended in 0.5 ml of T cell culture medium without IL-2, seeded into 48-well plates, and the culture supernatants were collected after 24, 48, and 72h of incubation. Subsequently, the supernatants were stored at -80℃. The secretion of IL-15/IL-15Rα was measured with Human IL-15 ELISA Kit (MultiSciences, Zhejiang, China) according to the instruction by a double--antibody sandwich enzyme-linked immunosorbent assay (ELISA).

**Detection of cell** **viability and proliferation by flow cytometry**

In the aforementioned experiments, supernatants were collected at 24, 48, and 72h for ELISA detection, while cells were stained and analyzed using flow cytometry to assess the viability and proliferation. T cells of NT, N, and N15 groups were stained with LIVE/DEAD Fixable Dead Cell Staining kit (LIVE/DEAD™ Fixable Near IR Viability Kit, L34992, Thermo, USA) according to the manufacturer’s instructions. Then the cells were suspended in 200μl/test and added with 25μl/test of CountBright™ absolute counting beads (Invitrogen) before testing on BD LSRFortessa. Gating strategy was shown in Supplementary Figure 5 and the absolute count of the live cells were calculated according to the following formula. The percentage and the absolute count of the live cells serves as indicators to assess the viability and proliferation, respectively.


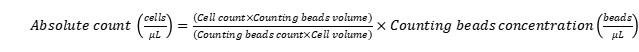


**RNA isolation and RNA-Seq**

On Day 13 post infection, 5 millions of T cells from NT, N and N15 groups were taken and co-cultured with B-LCLs at the a ratio of 2:1 for 8 hours under 37 °C and 5% CO2 (n=3). After RNA extraction with TRIzol reagent (Invitrogen, Carlsbad, USA) and quality inspection of purity (OD260/OD280: 1.8-2.4; OD260/OD230: 1.5-2.4) and integrity (RIN>7) by NanoPhotometer and Agilent 2100 Bioanalyzer, the total RNA was enriched with magnetic beads with oligo (dT) and fragmented and then used as the template to synthesize single-stranded cDNA; afterward, a two-strand synthesis reaction system was prepared to synthesize double-stranded cDNA; subsequently, the double-stranded cDNA underwent purification, recovery, sticky end repair, and the addition of the base "A" to the 3' end of the cDNA for adaptor ligation. Then, cDNA of the appropriate size was selected and amplified via PCR to construct cDNA libraries. Finally, the constructed libraries were qualified by an Agilent 2100 Bioanalyzer and Qubit and sequenced by an Illumina Novaseq™ 6000 (Guangzhou Huayin Health Technology Co., Ltd. Guangzhou, China) (PE150). The sequencing read length was 2×150 bp. Raw data were first filtered with fastp software (<https://github.com/OpenGene/fastp>) and subsequently mapped to the human reference genome (hg38) with the Hisat2 software. Gene expression abundance was estimated by RNASeq by expectation maximization (RSEM) and normalized to the number of fragments per kilobase of exon model per million mapped reads (FPKMs). The sample clustering was visualized using Principal Component Analysis (PCA). The differentially expressed genes (DEGs) were identified using the edgeR package in R software v3.8.1 with a threshold of log2-fold change >2.0 and *P*<0.05. All the DEGs were subjected to Gene Ontology (GO) and Kyoto Encyclopedia of Genes and Genomes (KEGG) pathway enrichment analysis using ClusterProfiler v3.8.1. GO terms or pathways with a corrected *P* value ≤ 0.05 were considered significantly enriched for DEGs. GO annotation results were analyzed by the Web Gene Ontology Annotation Plot (WEGO) software. Furthermore, we conducted Gene Set Enrichment Analysis (GSEA). The conventional method of enrichment analysis does not take into account changes in gene expression; its essence lies in the consistency of the distribution between different genes and random sampling outcomes. GSEA examines whether a set of genes is predominantly concentrated at either end of the sequence. If they are found at the top, it indicates an upward trend within the gene set; conversely, if they are located at the bottom, it signifies a downward trend. By incorporating these subtle expression changes, GSEA aims to achieve improved results.

Raw data were filtered with SOAPnuke v1.5.2. to get clean sequencing data by eliminating the adaptors or a large amount of unknown sequencing reads.

**Detection of *in vitro* cytokines by degranulation assay and intracellular staining (ICS)**

0.2×10^6^ target cells of B-LCLs/PBMCs and 1×10^6^ T cells of NT, N and N15 groups were suspended in 100μl of T cell culture medium without IL-2 respectively and co-cultured in a U-shaped 96-well plate. Then CD107a PE-Cy7 flow cytometry antibody (2μl/test) as well as BFA solution (2μl/test; containing 1:1000 Monensin from Biolegend and 1:1000 Brefeldin A from Biolegend) were added and the co-culture systems were incubated at 37°C in 5% CO2 for 5h, and subsequently collected for ICS. After the procedure of fixation/permeabilization, the cells were stained with antibodies (IFN-γ, TNF-α, IL-2 or Granzyme B for B-LCLs, and IFN-γ, IL-2 for PBMCs) for 30min at 2-8 ℃ before flow cytometry analysis (Supplementary Table 2).

**Detection of *in vitro*** **cytokines by ELISA**

The supernatants collected from the experiment of Figure 5C were applied for monitoring the cytokine secretion of IL-15, IL-2, IFN-γ, TNF-α, IL-6, IL-10 and GM-CSF at 6, 24, 48 and 72h after co-culturing by ELISA (The commercial kits used were listed in Supplementary Table 2, 50μl/test).

**Detection of *in vivo* cytokines by LEDGENDplex assay or ELISA**

On Day 21 after T cell treatment, mice of each group were sacrificed by breaking the neck to obtain the samples of peripheral blood (obtained by retro-orbital puncture). Then the blood samples were centrifuged at 400g×10min to get plasmas. A customized LEDGENDplex Human CD8/NK Panel (13-plex) with V-bottom Plate (741065, BioLegend, Japan) was used to detection of human IL-2, IL-4, IL-10, IL-6, IL-17A, TNF-α, sFas, sFasL, IFN-γ, Granzyme A, Granzyme B, Perforin and Granulysin in the plasmas of mice. According to the instructions of the manufacturer, the procedure include mainly the preparation of gradient standard and sample plasma (25μl/test, n=6), antigen capture, detection antibody incubation, washing, detection of gradient standard and parameter settings, detection of samples by flow cytometry and finally analyzing the data with the Qognit software (https://legendplex.qognit.com/; BioLegend, Japan). IL-15 in the plasma of mice was detected by the ELISA described above.

**Supplementary Tables.**

**Supplementary Table 1. Comparison of CD19 CAR-T or EBV scFv CAR-T with NKG2D CAR-T for treating EBV-PTLD**

|  | CD19 CAR-T vs NKG2D CAR-T | EBV scFv CAR-T vs NKG2D CAR-T |
| --- | --- | --- |
| Advantages | The expression of B-LCL showed a 99% CD19 positivity, with enhanced targeting efficiency. | It exhibits antiviral activity against Epstein-Barr virus (EBV) infected host cells, targeting the lysate protein (LMP-1) or glycoprotein (gp350). |
| Disadvantages | (1) The reconstitution of B-type hematopoiesis was suppressed by CD19 CAR-T;  (2) The immune system will experience evasion due to the loss of antigens;  (3) No promoted antiviral effect;  (4) The CD19 CAR-T therapy did not exhibit any impact on immunosuppressive cells, whereas NKG2D demonstrated the ability to specifically target myeloid-derived suppressor cells (MDSCs) and regulatory T cells (Tregs), as well as endothelial cells within the tumor microenvironment. | (1) The stability of protein expression exhibits periodic fluctuations;  (2) The scFv component is predominantly derived from mice or rabbits, whereas the NKG2D CAR structure originates from humans, thereby minimizing the potential for xenogenic immune response;  (3) Did not exhibit any impact on immunosuppressive cells. |

**Supplementary Table 2. Antibodies and kits used in flow cytometry, Western blot and ELISA**

| **Antigen** | **Clone** | **Fluorochrome** | **Source** | **Catalog number** |
| --- | --- | --- | --- | --- |
| MICA | #159227 | PE | R&D systems | FAB1300P-025 |
| MICA/B | 6D4 | APC | BioLegend | 320907 |
| ULBP-1 | #170818 | FITC | R&D systems | FAB1380G |
| ULBP-3 | #166510 | PE | R&D systems | FAB1517P |
| PE Human IgG1 Isotype Control | QA16A12 | PE | BioLegend | 403503 |
| APC Human IgG1 Isotype Control | QA16A12 | APC | BioLegend | 403505 |
| FITC Human IgG1 Isotype Control | QA16A12 | FITC | BioLegend | 403507 |
| **CAR and** **IL-15/IL-15Rα expression identified by Western blot analysis** | | | | |
| Rabbit Anti-Human CD3ζ | Polyclonal | / | Signalway Antibody | 32576 |
| Mouse His-Tag Antibody | 1B7G5 | / | Proteintech | 66005-1-lg |
| Rabbit Anti-Human IL-15 | Polyclonal | / | PeproTech | 500-P15-50ug |
| HRP Goat Anti Rabbit IgG(H+L) | / | / | Immunoway | RS0002 |
| HRP Goat Anti-Mouse IgG1 | / | / | abcam | ab97240 |
| **T cell phenotypic** | | | | |
| Anti-Human CD3 | UCHT1 | BV421 | BioLegend | 300434 |
| Anti-Human CD4 | OKT4 | PE | BioLegend | 317410 |
| Anti-Human CD8 | SK1 | PE-Cy5 | BioLegend | 344769 |
| Anti-Human CCR-7 | G043H7 | PE-Cy7 | BioLegend | 353225 |
| Anti-Human CD45RA | HI100 | APC | BioLegend | 983004 |
| Anti-Human CD25 | BC96 | PE-Cy5 | BioLegend | 302608 |
| Anti-Human CD69 | FN50 | APC | BioLegend | 310910 |
| Anti-Human PD-1 | EH12.2H7 | APC-Cy 7 | BioLegend | 329922 |
| Anti-Human LAG-3 | 11C3C65 | PE-Cy 7 | BioLegend | 369310 |
| Anti-Human TIM-3 | F38-2E2 | PerCP Cy5.5 | BioLegend | 345015 |
| **ICS** | | | | |
| Anti-human CD3 | UCHT1 | BV421 | BioLegend | 300434 |
| Anti-human CD107a | H4A3 | PE-Cy 7 | BioLegend | 328618 |
| Anti-human IFN-γ | 4S.B3 | PE | BioLegend | 502508 |
| Anti-human TNF-α | MAb11 | BV510 | BioLegend | 502950 |
| Anti-human IL-2 | MQ1-17H12 | APC | BioLegend | 500310 |
| Anti-human Granzyme B | QA16A02 | PE-Texas Red | BioLegend | 372216 |
| **ELISA** | | | | |
| IL-15 |  |  | MultiSciences | EK115-96 |
| IL-2 |  |  | MultiSciences | EK102HS-96 |
| IL-10 |  |  | MultiSciences | EK110HS-96 |
| IFN-γ |  |  | MultiSciences | EK180HS-96 |
| GM-CSF |  |  | MultiSciences | EK163HS-96 |
| TNF-α |  |  | MultiSciences | EK182HS-96 |
| IL-6 |  |  | MultiSciences | EK106HS-96 |
| **Evaluation of tumor burden and T cells in blood and tissues** | | | | |
| Anti-human CD3 | HIT3a | PerCP Cy5.5 | BioLegend | 300326 |
| Anti-human CD19 | HIB19 | PE | BioLegend | 302208 |

* Staining panel: Naïve/memory (CD3 BV421, FITC, CD4 PE, CD8 PE-Cy5, CCR-7 PE-Cy7, CD45RA-APC), activation (CD3 BV421, FITC, CD25 PE-Cy5, CD69 APC), exhaustion (CD3 BV421, FITC, PD-1 APC-Cy 7, LAG-3 PE-Cy 7, TIM-3 PerCP Cy5.5).

**Supplementary Table 3. IL-15/IL-15Rα expression detected by ELISA (pg/mL)**

| Group | 24h | 48h | 72h |
| --- | --- | --- | --- |
| NKG2D CAR-T cells | 5.39±6.90 | 6.14±7.37 | 1.75±2.36 |
| IL-15/IL-15Rα-NKG2D CAR-T cells | 2614.54±220.69 | 2686.78±98.08 | 2569.83±198.32 |
| Non-transduced T cells | 8.11±9.67 | 0.31±0.62 | 3.83±6.09 |

**Supplementary Table 4. RNA concentration, purity and integrity of T cell samples**

| Group | Sample | Concentration  (ng/μL) | Volume (μL) | 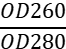 | 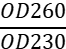 | Total RNA (μg) | RIN |
| --- | --- | --- | --- | --- | --- | --- | --- |
| N | AN | 143.52 | 38 | 2.02 | 0.55 | 5.45 | 9.6 |
|  | BN | 195.84 | 38 | 2.06 | 0.65 | 7.44 | 9.6 |
|  | CN | 149.72 | 38 | 2.09 | 1.51 | 5.69 | 9.7 |
| N15 | AN15 | 165.16 | 38 | 2.1 | 2.08 | 6.28 | 9.6 |
|  | BN15 | 292.84 | 38 | 2.08 | 1.29 | 11.13 | 8.9 |
|  | CN15 | 141.04 | 38 | 2.1 | 0.68 | 5.36 | 7.6 |
| NT | ANT | 178.76 | 38 | 2.09 | 2.01 | 6.79 | 9.6 |
|  | BNT | 188.24 | 38 | 2 | 0.43 | 7.15 | 10 |
|  | CNT | 136.28 | 38 | 1.88 | 0.52 | 5.18 | 10 |

**Supplementary Table 5. N_*vs*_NT GO Enrichment (Molecular Function)**

| GO ID | Description | GeneRatio (251) | BgRatio (1871) | P value | q value |
| --- | --- | --- | --- | --- | --- |
| [GO:0016301](file:///E:\\000000CAR%20NK\\00000%25E6%25AF%2595%25E4%25B8%259A%25E8%25AE%25BA%25E6%2596%2587\\%25E5%25AE%259E%25E9%25AA%258C%25E7%25BB%2593%25E6%259E%259CPPT%25E6%25B1%2587%25E6%2580%25BB\\9%20%25E8%25BD%25AC%25E5%25BD%2595%25E7%25BB%2584%25E6%25B5%258B%25E5%25BA%258F\\%25E7%25BB%2593%25E6%259E%259C%25E5%259B%25BE%25E7%2589%2587\\GO%25E5%2588%2586%25E6%259E%2590\\N_vs_NT.F.html#gene1) | kinase activity | 27 (10.76%) | 100 (5.34%) | 0.000153 | 0.008405 |
| [GO:0016772](file:///E:\\000000CAR%20NK\\00000%25E6%25AF%2595%25E4%25B8%259A%25E8%25AE%25BA%25E6%2596%2587\\%25E5%25AE%259E%25E9%25AA%258C%25E7%25BB%2593%25E6%259E%259CPPT%25E6%25B1%2587%25E6%2580%25BB\\9%20%25E8%25BD%25AC%25E5%25BD%2595%25E7%25BB%2584%25E6%25B5%258B%25E5%25BA%258F\\%25E7%25BB%2593%25E6%259E%259C%25E5%259B%25BE%25E7%2589%2587\\GO%25E5%2588%2586%25E6%259E%2590\\N_vs_NT.F.html#gene2) | transferase activity, transferring phosphorus-containing groups | 29 (11.55%) | 115 (6.15%) | 0.000319 | 0.008784 |
| [GO:0004871](file:///E:\\000000CAR%20NK\\00000%25E6%25AF%2595%25E4%25B8%259A%25E8%25AE%25BA%25E6%2596%2587\\%25E5%25AE%259E%25E9%25AA%258C%25E7%25BB%2593%25E6%259E%259CPPT%25E6%25B1%2587%25E6%2580%25BB\\9%20%25E8%25BD%25AC%25E5%25BD%2595%25E7%25BB%2584%25E6%25B5%258B%25E5%25BA%258F\\%25E7%25BB%2593%25E6%259E%259C%25E5%259B%25BE%25E7%2589%2587\\GO%25E5%2588%2586%25E6%259E%2590\\N_vs_NT.F.html#gene3) | signal transducer activity | 36 (14.34%) | 188 (10.05%) | 0.012573 | 0.230509 |
| [GO:0008289](file:///E:\\000000CAR%20NK\\00000%25E6%25AF%2595%25E4%25B8%259A%25E8%25AE%25BA%25E6%2596%2587\\%25E5%25AE%259E%25E9%25AA%258C%25E7%25BB%2593%25E6%259E%259CPPT%25E6%25B1%2587%25E6%2580%25BB\\9%20%25E8%25BD%25AC%25E5%25BD%2595%25E7%25BB%2584%25E6%25B5%258B%25E5%25BA%258F\\%25E7%25BB%2593%25E6%259E%259C%25E5%259B%25BE%25E7%2589%2587\\GO%25E5%2588%2586%25E6%259E%2590\\N_vs_NT.F.html#gene4) | lipid binding | 14 (5.58%) | 59 (3.15%) | 0.020378 | 0.280202 |
| [GO:0043167](file:///E:\\000000CAR%20NK\\00000%25E6%25AF%2595%25E4%25B8%259A%25E8%25AE%25BA%25E6%2596%2587\\%25E5%25AE%259E%25E9%25AA%258C%25E7%25BB%2593%25E6%259E%259CPPT%25E6%25B1%2587%25E6%2580%25BB\\9%20%25E8%25BD%25AC%25E5%25BD%2595%25E7%25BB%2584%25E6%25B5%258B%25E5%25BA%258F\\%25E7%25BB%2593%25E6%259E%259C%25E5%259B%25BE%25E7%2589%2587\\GO%25E5%2588%2586%25E6%259E%2590\\N_vs_NT.F.html#gene5) | ion binding | 98 (39.04%) | 629 (33.62%) | 0.030695 | 0.337647 |

**Supplementary Table 6. N_*vs*_NT GO Enrichment (Cellular Component)**

| GO ID | Description | GeneRatio (232) | BgRatio (1873) | P value | q value |
| --- | --- | --- | --- | --- | --- |
| [GO:0005886](file:///E:\\000000CAR%20NK\\00000%25E6%25AF%2595%25E4%25B8%259A%25E8%25AE%25BA%25E6%2596%2587\\%25E5%25AE%259E%25E9%25AA%258C%25E7%25BB%2593%25E6%259E%259CPPT%25E6%25B1%2587%25E6%2580%25BB\\9%20%25E8%25BD%25AC%25E5%25BD%2595%25E7%25BB%2584%25E6%25B5%258B%25E5%25BA%258F\\%25E7%25BB%2593%25E6%259E%259C%25E5%259B%25BE%25E7%2589%2587\\GO%25E5%2588%2586%25E6%259E%2590\\N_vs_NT.C.html#gene1) | plasma membrane | 80 (34.48%) | 450 (24.03%) | 0.000079 | 0.001378 |
| [GO:0016020](file:///E:\\000000CAR%20NK\\00000%25E6%25AF%2595%25E4%25B8%259A%25E8%25AE%25BA%25E6%2596%2587\\%25E5%25AE%259E%25E9%25AA%258C%25E7%25BB%2593%25E6%259E%259CPPT%25E6%25B1%2587%25E6%2580%25BB\\9%20%25E8%25BD%25AC%25E5%25BD%2595%25E7%25BB%2584%25E6%25B5%258B%25E5%25BA%258F\\%25E7%25BB%2593%25E6%259E%259C%25E5%259B%25BE%25E7%2589%2587\\GO%25E5%2588%2586%25E6%259E%2590\\N_vs_NT.C.html#gene2) | membrane | 80 (34.48%) | 450 (24.03%) | 0.000079 | 0.001378 |
| [GO:0071944](file:///E:\\000000CAR%20NK\\00000%25E6%25AF%2595%25E4%25B8%259A%25E8%25AE%25BA%25E6%2596%2587\\%25E5%25AE%259E%25E9%25AA%258C%25E7%25BB%2593%25E6%259E%259CPPT%25E6%25B1%2587%25E6%2580%25BB\\9%20%25E8%25BD%25AC%25E5%25BD%2595%25E7%25BB%2584%25E6%25B5%258B%25E5%25BA%258F\\%25E7%25BB%2593%25E6%259E%259C%25E5%259B%25BE%25E7%2589%2587\\GO%25E5%2588%2586%25E6%259E%2590\\N_vs_NT.C.html#gene3) | cell periphery | 80 (34.48%) | 450 (24.03%) | 0.000079 | 0.001378 |
| [GO:0005576](file:///E:\\000000CAR%20NK\\00000%25E6%25AF%2595%25E4%25B8%259A%25E8%25AE%25BA%25E6%2596%2587\\%25E5%25AE%259E%25E9%25AA%258C%25E7%25BB%2593%25E6%259E%259CPPT%25E6%25B1%2587%25E6%2580%25BB\\9%20%25E8%25BD%25AC%25E5%25BD%2595%25E7%25BB%2584%25E6%25B5%258B%25E5%25BA%258F\\%25E7%25BB%2593%25E6%259E%259C%25E5%259B%25BE%25E7%2589%2587\\GO%25E5%2588%2586%25E6%259E%2590\\N_vs_NT.C.html#gene4) | extracellular region | 50 (21.55%) | 247 (13.19%) | 0.000106 | 0.001378 |
| [GO:0044421](file:///E:\\000000CAR%20NK\\00000%25E6%25AF%2595%25E4%25B8%259A%25E8%25AE%25BA%25E6%2596%2587\\%25E5%25AE%259E%25E9%25AA%258C%25E7%25BB%2593%25E6%259E%259CPPT%25E6%25B1%2587%25E6%2580%25BB\\9%20%25E8%25BD%25AC%25E5%25BD%2595%25E7%25BB%2584%25E6%25B5%258B%25E5%25BA%258F\\%25E7%25BB%2593%25E6%259E%259C%25E5%259B%25BE%25E7%2589%2587\\GO%25E5%2588%2586%25E6%259E%2590\\N_vs_NT.C.html#gene5) | extracellular region part | 31 (13.36%) | 129 (6.89%) | 0.000109 | 0.001378 |
| [GO:0005615](file:///E:\\000000CAR%20NK\\00000%25E6%25AF%2595%25E4%25B8%259A%25E8%25AE%25BA%25E6%2596%2587\\%25E5%25AE%259E%25E9%25AA%258C%25E7%25BB%2593%25E6%259E%259CPPT%25E6%25B1%2587%25E6%2580%25BB\\9%20%25E8%25BD%25AC%25E5%25BD%2595%25E7%25BB%2584%25E6%25B5%258B%25E5%25BA%258F\\%25E7%25BB%2593%25E6%259E%259C%25E5%259B%25BE%25E7%2589%2587\\GO%25E5%2588%2586%25E6%259E%2590\\N_vs_NT.C.html#gene6) | extracellular space | 25 (10.78%) | 101 (5.39%) | 0.000327 | 0.003439 |
| [GO:0005578](file:///E:\\000000CAR%20NK\\00000%25E6%25AF%2595%25E4%25B8%259A%25E8%25AE%25BA%25E6%2596%2587\\%25E5%25AE%259E%25E9%25AA%258C%25E7%25BB%2593%25E6%259E%259CPPT%25E6%25B1%2587%25E6%2580%25BB\\9%20%25E8%25BD%25AC%25E5%25BD%2595%25E7%25BB%2584%25E6%25B5%258B%25E5%25BA%258F\\%25E7%25BB%2593%25E6%259E%259C%25E5%259B%25BE%25E7%2589%2587\\GO%25E5%2588%2586%25E6%259E%2590\\N_vs_NT.C.html#gene7) | proteinaceous extracellular matrix | 10 (4.31%) | 40 (2.14%) | 0.020219 | 0.159225 |
| [GO:0031012](file:///E:\\000000CAR%20NK\\00000%25E6%25AF%2595%25E4%25B8%259A%25E8%25AE%25BA%25E6%2596%2587\\%25E5%25AE%259E%25E9%25AA%258C%25E7%25BB%2593%25E6%259E%259CPPT%25E6%25B1%2587%25E6%2580%25BB\\9%20%25E8%25BD%25AC%25E5%25BD%2595%25E7%25BB%2584%25E6%25B5%258B%25E5%25BA%258F\\%25E7%25BB%2593%25E6%259E%259C%25E5%259B%25BE%25E7%2589%2587\\GO%25E5%2588%2586%25E6%259E%2590\\N_vs_NT.C.html#gene8) | extracellular matrix | 10 (4.31%) | 40 (2.14%) | 0.020219 | 0.159225 |
| [GO:0005783](file:///E:\\000000CAR%20NK\\00000%25E6%25AF%2595%25E4%25B8%259A%25E8%25AE%25BA%25E6%2596%2587\\%25E5%25AE%259E%25E9%25AA%258C%25E7%25BB%2593%25E6%259E%259CPPT%25E6%25B1%2587%25E6%2580%25BB\\9%20%25E8%25BD%25AC%25E5%25BD%2595%25E7%25BB%2584%25E6%25B5%258B%25E5%25BA%258F\\%25E7%25BB%2593%25E6%259E%259C%25E5%259B%25BE%25E7%2589%2587\\GO%25E5%2588%2586%25E6%259E%2590\\N_vs_NT.C.html#gene9) | endoplasmic reticulum | 28 (12.07%) | 162 (8.65%) | 0.035758 | 0.232256 |
| [GO:0005829](file:///E:\\000000CAR%20NK\\00000%25E6%25AF%2595%25E4%25B8%259A%25E8%25AE%25BA%25E6%2596%2587\\%25E5%25AE%259E%25E9%25AA%258C%25E7%25BB%2593%25E6%259E%259CPPT%25E6%25B1%2587%25E6%2580%25BB\\9%20%25E8%25BD%25AC%25E5%25BD%2595%25E7%25BB%2584%25E6%25B5%258B%25E5%25BA%258F\\%25E7%25BB%2593%25E6%259E%259C%25E5%259B%25BE%25E7%2589%2587\\GO%25E5%2588%2586%25E6%259E%2590\\N_vs_NT.C.html#gene10) | cytosol | 48 (20.69%) | 306 (16.34%) | 0.036866 | 0.232256 |

**Supplementary Table 7. N_*vs*_NT GO Enrichment (Biological Process)**

| GO ID | Description | GeneRatio (223) | BgRatio  (1701) | p value | q value |
| --- | --- | --- | --- | --- | --- |
| [GO:0040011](file:///E:\\000000CAR%20NK\\00000%25E6%25AF%2595%25E4%25B8%259A%25E8%25AE%25BA%25E6%2596%2587\\%25E5%25AE%259E%25E9%25AA%258C%25E7%25BB%2593%25E6%259E%259CPPT%25E6%25B1%2587%25E6%2580%25BB\\9%20%25E8%25BD%25AC%25E5%25BD%2595%25E7%25BB%2584%25E6%25B5%258B%25E5%25BA%258F\\%25E7%25BB%2593%25E6%259E%259C%25E5%259B%25BE%25E7%2589%2587\\GO%25E5%2588%2586%25E6%259E%2590\\N_vs_NT.P.html#gene1) | locomotion | 45 (20.18%) | 149 (8.76%) | 0 | 0.000001 |
| [GO:0032502](file:///E:\\000000CAR%20NK\\00000%25E6%25AF%2595%25E4%25B8%259A%25E8%25AE%25BA%25E6%2596%2587\\%25E5%25AE%259E%25E9%25AA%258C%25E7%25BB%2593%25E6%259E%259CPPT%25E6%25B1%2587%25E6%2580%25BB\\9%20%25E8%25BD%25AC%25E5%25BD%2595%25E7%25BB%2584%25E6%25B5%258B%25E5%25BA%258F\\%25E7%25BB%2593%25E6%259E%259C%25E5%259B%25BE%25E7%2589%2587\\GO%25E5%2588%2586%25E6%259E%2590\\N_vs_NT.P.html#gene2) | developmental process | 99 (44.39%) | 504 (29.63%) | 0 | 0.000015 |
| [GO:0007155](file:///E:\\000000CAR%20NK\\00000%25E6%25AF%2595%25E4%25B8%259A%25E8%25AE%25BA%25E6%2596%2587\\%25E5%25AE%259E%25E9%25AA%258C%25E7%25BB%2593%25E6%259E%259CPPT%25E6%25B1%2587%25E6%2580%25BB\\9%20%25E8%25BD%25AC%25E5%25BD%2595%25E7%25BB%2584%25E6%25B5%258B%25E5%25BA%258F\\%25E7%25BB%2593%25E6%259E%259C%25E5%259B%25BE%25E7%2589%2587\\GO%25E5%2588%2586%25E6%259E%2590\\N_vs_NT.P.html#gene3) | cell adhesion | 31 (13.9%) | 96 (5.64%) | 0 | 0.000015 |
| [GO:0022610](file:///E:\\000000CAR%20NK\\00000%25E6%25AF%2595%25E4%25B8%259A%25E8%25AE%25BA%25E6%2596%2587\\%25E5%25AE%259E%25E9%25AA%258C%25E7%25BB%2593%25E6%259E%259CPPT%25E6%25B1%2587%25E6%2580%25BB\\9%20%25E8%25BD%25AC%25E5%25BD%2595%25E7%25BB%2584%25E6%25B5%258B%25E5%25BA%258F\\%25E7%25BB%2593%25E6%259E%259C%25E5%259B%25BE%25E7%2589%2587\\GO%25E5%2588%2586%25E6%259E%2590\\N_vs_NT.P.html#gene4) | biological adhesion | 31 (13.9%) | 96 (5.64%) | 0 | 0.000015 |
| [GO:0048856](file:///E:\\000000CAR%20NK\\00000%25E6%25AF%2595%25E4%25B8%259A%25E8%25AE%25BA%25E6%2596%2587\\%25E5%25AE%259E%25E9%25AA%258C%25E7%25BB%2593%25E6%259E%259CPPT%25E6%25B1%2587%25E6%2580%25BB\\9%20%25E8%25BD%25AC%25E5%25BD%2595%25E7%25BB%2584%25E6%25B5%258B%25E5%25BA%258F\\%25E7%25BB%2593%25E6%259E%259C%25E5%259B%25BE%25E7%2589%2587\\GO%25E5%2588%2586%25E6%259E%2590\\N_vs_NT.P.html#gene5) | anatomical structure development | 92 (41.26%) | 463 (27.22%) | 0.000001 | 0.000023 |
| [GO:0009653](file:///E:\\000000CAR%20NK\\00000%25E6%25AF%2595%25E4%25B8%259A%25E8%25AE%25BA%25E6%2596%2587\\%25E5%25AE%259E%25E9%25AA%258C%25E7%25BB%2593%25E6%259E%259CPPT%25E6%25B1%2587%25E6%2580%25BB\\9%20%25E8%25BD%25AC%25E5%25BD%2595%25E7%25BB%2584%25E6%25B5%258B%25E5%25BA%258F\\%25E7%25BB%2593%25E6%259E%259C%25E5%259B%25BE%25E7%2589%2587\\GO%25E5%2588%2586%25E6%259E%2590\\N_vs_NT.P.html#gene6) | anatomical structure morphogenesis | 42 (18.83%) | 170 (9.99%) | 0.00001 | 0.000223 |
| [GO:0006928](file:///E:\\000000CAR%20NK\\00000%25E6%25AF%2595%25E4%25B8%259A%25E8%25AE%25BA%25E6%2596%2587\\%25E5%25AE%259E%25E9%25AA%258C%25E7%25BB%2593%25E6%259E%259CPPT%25E6%25B1%2587%25E6%2580%25BB\\9%20%25E8%25BD%25AC%25E5%25BD%2595%25E7%25BB%2584%25E6%25B5%258B%25E5%25BA%258F\\%25E7%25BB%2593%25E6%259E%259C%25E5%259B%25BE%25E7%2589%2587\\GO%25E5%2588%2586%25E6%259E%2590\\N_vs_NT.P.html#gene7) | movement of cell or subcellular component | 31 (13.9%) | 111 (6.53%) | 0.000013 | 0.000223 |
| [GO:0048870](file:///E:\\000000CAR%20NK\\00000%25E6%25AF%2595%25E4%25B8%259A%25E8%25AE%25BA%25E6%2596%2587\\%25E5%25AE%259E%25E9%25AA%258C%25E7%25BB%2593%25E6%259E%259CPPT%25E6%25B1%2587%25E6%2580%25BB\\9%20%25E8%25BD%25AC%25E5%25BD%2595%25E7%25BB%2584%25E6%25B5%258B%25E5%25BA%258F\\%25E7%25BB%2593%25E6%259E%259C%25E5%259B%25BE%25E7%2589%2587\\GO%25E5%2588%2586%25E6%259E%2590\\N_vs_NT.P.html#gene8) | cell motility | 31 (13.9%) | 111 (6.53%) | 0.000013 | 0.000223 |
| [GO:0051674](file:///E:\\000000CAR%20NK\\00000%25E6%25AF%2595%25E4%25B8%259A%25E8%25AE%25BA%25E6%2596%2587\\%25E5%25AE%259E%25E9%25AA%258C%25E7%25BB%2593%25E6%259E%259CPPT%25E6%25B1%2587%25E6%2580%25BB\\9%20%25E8%25BD%25AC%25E5%25BD%2595%25E7%25BB%2584%25E6%25B5%258B%25E5%25BA%258F\\%25E7%25BB%2593%25E6%259E%259C%25E5%259B%25BE%25E7%2589%2587\\GO%25E5%2588%2586%25E6%259E%2590\\N_vs_NT.P.html#gene9) | localization of cell | 31 (13.9%) | 111 (6.53%) | 0.000013 | 0.000223 |
| [GO:0030154](file:///E:\\000000CAR%20NK\\00000%25E6%25AF%2595%25E4%25B8%259A%25E8%25AE%25BA%25E6%2596%2587\\%25E5%25AE%259E%25E9%25AA%258C%25E7%25BB%2593%25E6%259E%259CPPT%25E6%25B1%2587%25E6%2580%25BB\\9%20%25E8%25BD%25AC%25E5%25BD%2595%25E7%25BB%2584%25E6%25B5%258B%25E5%25BA%258F\\%25E7%25BB%2593%25E6%259E%259C%25E5%259B%25BE%25E7%2589%2587\\GO%25E5%2588%2586%25E6%259E%2590\\N_vs_NT.P.html#gene10) | cell differentiation | 67 (30.04%) | 326 (19.17%) | 0.000017 | 0.000256 |
| [GO:0048869](file:///E:\\000000CAR%20NK\\00000%25E6%25AF%2595%25E4%25B8%259A%25E8%25AE%25BA%25E6%2596%2587\\%25E5%25AE%259E%25E9%25AA%258C%25E7%25BB%2593%25E6%259E%259CPPT%25E6%25B1%2587%25E6%2580%25BB\\9%20%25E8%25BD%25AC%25E5%25BD%2595%25E7%25BB%2584%25E6%25B5%258B%25E5%25BA%258F\\%25E7%25BB%2593%25E6%259E%259C%25E5%259B%25BE%25E7%2589%2587\\GO%25E5%2588%2586%25E6%259E%2590\\N_vs_NT.P.html#gene11) | cellular developmental process | 70 (31.39%) | 346 (20.34%) | 0.000018 | 0.000256 |
| [GO:0044763](file:///E:\\000000CAR%20NK\\00000%25E6%25AF%2595%25E4%25B8%259A%25E8%25AE%25BA%25E6%2596%2587\\%25E5%25AE%259E%25E9%25AA%258C%25E7%25BB%2593%25E6%259E%259CPPT%25E6%25B1%2587%25E6%2580%25BB\\9%20%25E8%25BD%25AC%25E5%25BD%2595%25E7%25BB%2584%25E6%25B5%258B%25E5%25BA%258F\\%25E7%25BB%2593%25E6%259E%259C%25E5%259B%25BE%25E7%2589%2587\\GO%25E5%2588%2586%25E6%259E%2590\\N_vs_NT.P.html#gene12) | single-organism cellular process | 150 (67.26%) | 928 (54.56%) | 0.000025 | 0.000315 |
| [GO:0044699](file:///E:\\000000CAR%20NK\\00000%25E6%25AF%2595%25E4%25B8%259A%25E8%25AE%25BA%25E6%2596%2587\\%25E5%25AE%259E%25E9%25AA%258C%25E7%25BB%2593%25E6%259E%259CPPT%25E6%25B1%2587%25E6%2580%25BB\\9%20%25E8%25BD%25AC%25E5%25BD%2595%25E7%25BB%2584%25E6%25B5%258B%25E5%25BA%258F\\%25E7%25BB%2593%25E6%259E%259C%25E5%259B%25BE%25E7%2589%2587\\GO%25E5%2588%2586%25E6%259E%2590\\N_vs_NT.P.html#gene13) | single-organism process | 170 (76.23%) | 1097 (64.49%) | 0.000038 | 0.000456 |
| [GO:0044767](file:///E:\\000000CAR%20NK\\00000%25E6%25AF%2595%25E4%25B8%259A%25E8%25AE%25BA%25E6%2596%2587\\%25E5%25AE%259E%25E9%25AA%258C%25E7%25BB%2593%25E6%259E%259CPPT%25E6%25B1%2587%25E6%2580%25BB\\9%20%25E8%25BD%25AC%25E5%25BD%2595%25E7%25BB%2584%25E6%25B5%258B%25E5%25BA%258F\\%25E7%25BB%2593%25E6%259E%259C%25E5%259B%25BE%25E7%2589%2587\\GO%25E5%2588%2586%25E6%259E%2590\\N_vs_NT.P.html#gene14) | single-organism developmental process | 75 (33.63%) | 391 (22.99%) | 0.000063 | 0.000693 |

**Supplementary Table 8. N15_*vs*_NT GO Enrichment (Molecular Function)**

| GO ID | Description | GeneRatio (228) | BgRatio (1871) | p value | q value |
| --- | --- | --- | --- | --- | --- |
| [GO:0016772](file:///E:\\000000CAR%20NK\\00000%25E6%25AF%2595%25E4%25B8%259A%25E8%25AE%25BA%25E6%2596%2587\\%25E5%25AE%259E%25E9%25AA%258C%25E7%25BB%2593%25E6%259E%259CPPT%25E6%25B1%2587%25E6%2580%25BB\\9%20%25E8%25BD%25AC%25E5%25BD%2595%25E7%25BB%2584%25E6%25B5%258B%25E5%25BA%258F\\%25E7%25BB%2593%25E6%259E%259C%25E5%259B%25BE%25E7%2589%2587\\GO%25E5%2588%2586%25E6%259E%2590\\N15_vs_NT.F.html#gene1) | transferase activity, transferring phosphorus-containing groups | 27 (11.84%) | 115 (6.15%) | 0.000361 | 0.015492 |
| [GO:0016301](file:///E:\\000000CAR%20NK\\00000%25E6%25AF%2595%25E4%25B8%259A%25E8%25AE%25BA%25E6%2596%2587\\%25E5%25AE%259E%25E9%25AA%258C%25E7%25BB%2593%25E6%259E%259CPPT%25E6%25B1%2587%25E6%2580%25BB\\9%20%25E8%25BD%25AC%25E5%25BD%2595%25E7%25BB%2584%25E6%25B5%258B%25E5%25BA%258F\\%25E7%25BB%2593%25E6%259E%259C%25E5%259B%25BE%25E7%2589%2587\\GO%25E5%2588%2586%25E6%259E%2590\\N15_vs_NT.F.html#gene2) | kinase activity | 24 (10.53%) | 100 (5.34%) | 0.000553 | 0.015492 |
| [GO:0008289](file:///E:\\000000CAR%20NK\\00000%25E6%25AF%2595%25E4%25B8%259A%25E8%25AE%25BA%25E6%2596%2587\\%25E5%25AE%259E%25E9%25AA%258C%25E7%25BB%2593%25E6%259E%259CPPT%25E6%25B1%2587%25E6%2580%25BB\\9%20%25E8%25BD%25AC%25E5%25BD%2595%25E7%25BB%2584%25E6%25B5%258B%25E5%25BA%258F\\%25E7%25BB%2593%25E6%259E%259C%25E5%259B%25BE%25E7%2589%2587\\GO%25E5%2588%2586%25E6%259E%2590\\N15_vs_NT.F.html#gene3) | lipid binding | 15 (6.58%) | 59 (3.15%) | 0.003452 | 0.064433 |
| [GO:0016740](file:///E:\\000000CAR%20NK\\00000%25E6%25AF%2595%25E4%25B8%259A%25E8%25AE%25BA%25E6%2596%2587\\%25E5%25AE%259E%25E9%25AA%258C%25E7%25BB%2593%25E6%259E%259CPPT%25E6%25B1%2587%25E6%2580%25BB\\9%20%25E8%25BD%25AC%25E5%25BD%2595%25E7%25BB%2584%25E6%25B5%258B%25E5%25BA%258F\\%25E7%25BB%2593%25E6%259E%259C%25E5%259B%25BE%25E7%2589%2587\\GO%25E5%2588%2586%25E6%259E%2590\\N15_vs_NT.F.html#gene4) | transferase activity | 34 (14.91%) | 183 (9.78%) | 0.005455 | 0.076369 |

**Supplementary Table 9. N15_*vs*_NT GO Enrichment (Cellular Component)**

| GO ID | Description | GeneRatio (220) | BgRatio  (1873) | p value | q value |
| --- | --- | --- | --- | --- | --- |
| [GO:0005886](file:///E:\\000000CAR%20NK\\00000%25E6%25AF%2595%25E4%25B8%259A%25E8%25AE%25BA%25E6%2596%2587\\%25E5%25AE%259E%25E9%25AA%258C%25E7%25BB%2593%25E6%259E%259CPPT%25E6%25B1%2587%25E6%2580%25BB\\9%20%25E8%25BD%25AC%25E5%25BD%2595%25E7%25BB%2584%25E6%25B5%258B%25E5%25BA%258F\\%25E7%25BB%2593%25E6%259E%259C%25E5%259B%25BE%25E7%2589%2587\\GO%25E5%2588%2586%25E6%259E%2590\\N15_vs_NT.C.html#gene1) | plasma membrane | 76 (34.55%) | 450 (24.03%) | 0.000115 | 0.002425 |
| [GO:0016020](file:///E:\\000000CAR%20NK\\00000%25E6%25AF%2595%25E4%25B8%259A%25E8%25AE%25BA%25E6%2596%2587\\%25E5%25AE%259E%25E9%25AA%258C%25E7%25BB%2593%25E6%259E%259CPPT%25E6%25B1%2587%25E6%2580%25BB\\9%20%25E8%25BD%25AC%25E5%25BD%2595%25E7%25BB%2584%25E6%25B5%258B%25E5%25BA%258F\\%25E7%25BB%2593%25E6%259E%259C%25E5%259B%25BE%25E7%2589%2587\\GO%25E5%2588%2586%25E6%259E%2590\\N15_vs_NT.C.html#gene2) | membrane | 76 (34.55%) | 450 (24.03%) | 0.000115 | 0.002425 |
| [GO:0071944](file:///E:\\000000CAR%20NK\\00000%25E6%25AF%2595%25E4%25B8%259A%25E8%25AE%25BA%25E6%2596%2587\\%25E5%25AE%259E%25E9%25AA%258C%25E7%25BB%2593%25E6%259E%259CPPT%25E6%25B1%2587%25E6%2580%25BB\\9%20%25E8%25BD%25AC%25E5%25BD%2595%25E7%25BB%2584%25E6%25B5%258B%25E5%25BA%258F\\%25E7%25BB%2593%25E6%259E%259C%25E5%259B%25BE%25E7%2589%2587\\GO%25E5%2588%2586%25E6%259E%2590\\N15_vs_NT.C.html#gene3) | cell periphery | 76 (34.55%) | 450 (24.03%) | 0.000115 | 0.002425 |
| [GO:0005737](file:///E:\\000000CAR%20NK\\00000%25E6%25AF%2595%25E4%25B8%259A%25E8%25AE%25BA%25E6%2596%2587\\%25E5%25AE%259E%25E9%25AA%258C%25E7%25BB%2593%25E6%259E%259CPPT%25E6%25B1%2587%25E6%2580%25BB\\9%20%25E8%25BD%25AC%25E5%25BD%2595%25E7%25BB%2584%25E6%25B5%258B%25E5%25BA%258F\\%25E7%25BB%2593%25E6%259E%259C%25E5%259B%25BE%25E7%2589%2587\\GO%25E5%2588%2586%25E6%259E%2590\\N15_vs_NT.C.html#gene4) | cytoplasm | 143 (65%) | 1069 (57.07%) | 0.006699 | 0.105513 |
| [GO:0044444](file:///E:\\000000CAR%20NK\\00000%25E6%25AF%2595%25E4%25B8%259A%25E8%25AE%25BA%25E6%2596%2587\\%25E5%25AE%259E%25E9%25AA%258C%25E7%25BB%2593%25E6%259E%259CPPT%25E6%25B1%2587%25E6%2580%25BB\\9%20%25E8%25BD%25AC%25E5%25BD%2595%25E7%25BB%2584%25E6%25B5%258B%25E5%25BA%258F\\%25E7%25BB%2593%25E6%259E%259C%25E5%259B%25BE%25E7%2589%2587\\GO%25E5%2588%2586%25E6%259E%2590\\N15_vs_NT.C.html#gene5) | cytoplasmic part | 104 (47.27%) | 745 (39.78%) | 0.009867 | 0.109363 |
| [GO:0005615](file:///E:\\000000CAR%20NK\\00000%25E6%25AF%2595%25E4%25B8%259A%25E8%25AE%25BA%25E6%2596%2587\\%25E5%25AE%259E%25E9%25AA%258C%25E7%25BB%2593%25E6%259E%259CPPT%25E6%25B1%2587%25E6%2580%25BB\\9%20%25E8%25BD%25AC%25E5%25BD%2595%25E7%25BB%2584%25E6%25B5%258B%25E5%25BA%258F\\%25E7%25BB%2593%25E6%259E%259C%25E5%259B%25BE%25E7%2589%2587\\GO%25E5%2588%2586%25E6%259E%2590\\N15_vs_NT.C.html#gene6) | extracellular space | 20 (9.09%) | 101 (5.39%) | 0.010987 | 0.109363 |
| [GO:0044421](file:///E:\\000000CAR%20NK\\00000%25E6%25AF%2595%25E4%25B8%259A%25E8%25AE%25BA%25E6%2596%2587\\%25E5%25AE%259E%25E9%25AA%258C%25E7%25BB%2593%25E6%259E%259CPPT%25E6%25B1%2587%25E6%2580%25BB\\9%20%25E8%25BD%25AC%25E5%25BD%2595%25E7%25BB%2584%25E6%25B5%258B%25E5%25BA%258F\\%25E7%25BB%2593%25E6%259E%259C%25E5%259B%25BE%25E7%2589%2587\\GO%25E5%2588%2586%25E6%259E%2590\\N15_vs_NT.C.html#gene7) | extracellular region part | 24 (10.91%) | 129 (6.89%) | 0.012151 | 0.109363 |
| [GO:0005829](file:///E:\\000000CAR%20NK\\00000%25E6%25AF%2595%25E4%25B8%259A%25E8%25AE%25BA%25E6%2596%2587\\%25E5%25AE%259E%25E9%25AA%258C%25E7%25BB%2593%25E6%259E%259CPPT%25E6%25B1%2587%25E6%2580%25BB\\9%20%25E8%25BD%25AC%25E5%25BD%2595%25E7%25BB%2584%25E6%25B5%258B%25E5%25BA%258F\\%25E7%25BB%2593%25E6%259E%259C%25E5%259B%25BE%25E7%2589%2587\\GO%25E5%2588%2586%25E6%259E%2590\\N15_vs_NT.C.html#gene8) | cytosol | 48 (21.82%) | 306 (16.34%) | 0.014412 | 0.113494 |
| [GO:0005576](file:///E:\\000000CAR%20NK\\00000%25E6%25AF%2595%25E4%25B8%259A%25E8%25AE%25BA%25E6%2596%2587\\%25E5%25AE%259E%25E9%25AA%258C%25E7%25BB%2593%25E6%259E%259CPPT%25E6%25B1%2587%25E6%2580%25BB\\9%20%25E8%25BD%25AC%25E5%25BD%2595%25E7%25BB%2584%25E6%25B5%258B%25E5%25BA%258F\\%25E7%25BB%2593%25E6%259E%259C%25E5%259B%25BE%25E7%2589%2587\\GO%25E5%2588%2586%25E6%259E%2590\\N15_vs_NT.C.html#gene9) | extracellular region | 39 (17.73%) | 247 (13.19%) | 0.024969 | 0.174785 |

**Supplementary Table 10. N15_*vs*_NT GO Enrichment (Biological Process)**

| GO ID | Description | GeneRatio (203) | BgRatio (1701) | p value | q value |
| --- | --- | --- | --- | --- | --- |
| [GO:0040011](file:///E:\\000000CAR%20NK\\00000%25E6%25AF%2595%25E4%25B8%259A%25E8%25AE%25BA%25E6%2596%2587\\%25E5%25AE%259E%25E9%25AA%258C%25E7%25BB%2593%25E6%259E%259CPPT%25E6%25B1%2587%25E6%2580%25BB\\9%20%25E8%25BD%25AC%25E5%25BD%2595%25E7%25BB%2584%25E6%25B5%258B%25E5%25BA%258F\\%25E7%25BB%2593%25E6%259E%259C%25E5%259B%25BE%25E7%2589%2587\\GO%25E5%2588%2586%25E6%259E%2590\\N15_vs_NT.P.html#gene1) | locomotion | 38 (18.72%) | 149 (8.76%) | 0.000001 | 0.000182 |
| [GO:0006928](file:///E:\\000000CAR%20NK\\00000%25E6%25AF%2595%25E4%25B8%259A%25E8%25AE%25BA%25E6%2596%2587\\%25E5%25AE%259E%25E9%25AA%258C%25E7%25BB%2593%25E6%259E%259CPPT%25E6%25B1%2587%25E6%2580%25BB\\9%20%25E8%25BD%25AC%25E5%25BD%2595%25E7%25BB%2584%25E6%25B5%258B%25E5%25BA%258F\\%25E7%25BB%2593%25E6%259E%259C%25E5%259B%25BE%25E7%2589%2587\\GO%25E5%2588%2586%25E6%259E%2590\\N15_vs_NT.P.html#gene2) | movement of cell or subcellular component | 29 (14.29%) | 111 (6.53%) | 0.000016 | 0.000568 |
| [GO:0048870](file:///E:\\000000CAR%20NK\\00000%25E6%25AF%2595%25E4%25B8%259A%25E8%25AE%25BA%25E6%2596%2587\\%25E5%25AE%259E%25E9%25AA%258C%25E7%25BB%2593%25E6%259E%259CPPT%25E6%25B1%2587%25E6%2580%25BB\\9%20%25E8%25BD%25AC%25E5%25BD%2595%25E7%25BB%2584%25E6%25B5%258B%25E5%25BA%258F\\%25E7%25BB%2593%25E6%259E%259C%25E5%259B%25BE%25E7%2589%2587\\GO%25E5%2588%2586%25E6%259E%2590\\N15_vs_NT.P.html#gene3) | cell motility | 29 (14.29%) | 111 (6.53%) | 0.000016 | 0.000568 |
| [GO:0051674](file:///E:\\000000CAR%20NK\\00000%25E6%25AF%2595%25E4%25B8%259A%25E8%25AE%25BA%25E6%2596%2587\\%25E5%25AE%259E%25E9%25AA%258C%25E7%25BB%2593%25E6%259E%259CPPT%25E6%25B1%2587%25E6%2580%25BB\\9%20%25E8%25BD%25AC%25E5%25BD%2595%25E7%25BB%2584%25E6%25B5%258B%25E5%25BA%258F\\%25E7%25BB%2593%25E6%259E%259C%25E5%259B%25BE%25E7%2589%2587\\GO%25E5%2588%2586%25E6%259E%2590\\N15_vs_NT.P.html#gene4) | localization of cell | 29 (14.29%) | 111 (6.53%) | 0.000016 | 0.000568 |
| [GO:0007165](file:///E:\\000000CAR%20NK\\00000%25E6%25AF%2595%25E4%25B8%259A%25E8%25AE%25BA%25E6%2596%2587\\%25E5%25AE%259E%25E9%25AA%258C%25E7%25BB%2593%25E6%259E%259CPPT%25E6%25B1%2587%25E6%2580%25BB\\9%20%25E8%25BD%25AC%25E5%25BD%2595%25E7%25BB%2584%25E6%25B5%258B%25E5%25BA%258F\\%25E7%25BB%2593%25E6%259E%259C%25E5%259B%25BE%25E7%2589%2587\\GO%25E5%2588%2586%25E6%259E%2590\\N15_vs_NT.P.html#gene5) | signal transduction | 93 (45.81%) | 559 (32.86%) | 0.000029 | 0.000568 |
| [GO:0050789](file:///E:\\000000CAR%20NK\\00000%25E6%25AF%2595%25E4%25B8%259A%25E8%25AE%25BA%25E6%2596%2587\\%25E5%25AE%259E%25E9%25AA%258C%25E7%25BB%2593%25E6%259E%259CPPT%25E6%25B1%2587%25E6%2580%25BB\\9%20%25E8%25BD%25AC%25E5%25BD%2595%25E7%25BB%2584%25E6%25B5%258B%25E5%25BA%258F\\%25E7%25BB%2593%25E6%259E%259C%25E5%259B%25BE%25E7%2589%2587\\GO%25E5%2588%2586%25E6%259E%2590\\N15_vs_NT.P.html#gene6) | regulation of biological process | 93 (45.81%) | 559 (32.86%) | 0.000029 | 0.000568 |
| [GO:0050794](file:///E:\\000000CAR%20NK\\00000%25E6%25AF%2595%25E4%25B8%259A%25E8%25AE%25BA%25E6%2596%2587\\%25E5%25AE%259E%25E9%25AA%258C%25E7%25BB%2593%25E6%259E%259CPPT%25E6%25B1%2587%25E6%2580%25BB\\9%20%25E8%25BD%25AC%25E5%25BD%2595%25E7%25BB%2584%25E6%25B5%258B%25E5%25BA%258F\\%25E7%25BB%2593%25E6%259E%259C%25E5%259B%25BE%25E7%2589%2587\\GO%25E5%2588%2586%25E6%259E%2590\\N15_vs_NT.P.html#gene7) | regulation of cellular process | 93 (45.81%) | 559 (32.86%) | 0.000029 | 0.000568 |
| [GO:0051716](file:///E:\\000000CAR%20NK\\00000%25E6%25AF%2595%25E4%25B8%259A%25E8%25AE%25BA%25E6%2596%2587\\%25E5%25AE%259E%25E9%25AA%258C%25E7%25BB%2593%25E6%259E%259CPPT%25E6%25B1%2587%25E6%2580%25BB\\9%20%25E8%25BD%25AC%25E5%25BD%2595%25E7%25BB%2584%25E6%25B5%258B%25E5%25BA%258F\\%25E7%25BB%2593%25E6%259E%259C%25E5%259B%25BE%25E7%2589%2587\\GO%25E5%2588%2586%25E6%259E%2590\\N15_vs_NT.P.html#gene8) | cellular response to stimulus | 93 (45.81%) | 559 (32.86%) | 0.000029 | 0.000568 |
| [GO:0002376](file:///E:\\000000CAR%20NK\\00000%25E6%25AF%2595%25E4%25B8%259A%25E8%25AE%25BA%25E6%2596%2587\\%25E5%25AE%259E%25E9%25AA%258C%25E7%25BB%2593%25E6%259E%259CPPT%25E6%25B1%2587%25E6%2580%25BB\\9%20%25E8%25BD%25AC%25E5%25BD%2595%25E7%25BB%2584%25E6%25B5%258B%25E5%25BA%258F\\%25E7%25BB%2593%25E6%259E%259C%25E5%259B%25BE%25E7%2589%2587\\GO%25E5%2588%2586%25E6%259E%2590\\N15_vs_NT.P.html#gene9) | immune system process | 48 (23.65%) | 235 (13.82%) | 0.000037 | 0.000623 |
| [GO:0044699](file:///E:\\000000CAR%20NK\\00000%25E6%25AF%2595%25E4%25B8%259A%25E8%25AE%25BA%25E6%2596%2587\\%25E5%25AE%259E%25E9%25AA%258C%25E7%25BB%2593%25E6%259E%259CPPT%25E6%25B1%2587%25E6%2580%25BB\\9%20%25E8%25BD%25AC%25E5%25BD%2595%25E7%25BB%2584%25E6%25B5%258B%25E5%25BA%258F\\%25E7%25BB%2593%25E6%259E%259C%25E5%259B%25BE%25E7%2589%2587\\GO%25E5%2588%2586%25E6%259E%2590\\N15_vs_NT.P.html#gene10) | single-organism process | 156 (76.85%) | 1097(64.49%) | 0.000039 | 0.000623 |
| [GO:0044763](file:///E:\\000000CAR%20NK\\00000%25E6%25AF%2595%25E4%25B8%259A%25E8%25AE%25BA%25E6%2596%2587\\%25E5%25AE%259E%25E9%25AA%258C%25E7%25BB%2593%25E6%259E%259CPPT%25E6%25B1%2587%25E6%2580%25BB\\9%20%25E8%25BD%25AC%25E5%25BD%2595%25E7%25BB%2584%25E6%25B5%258B%25E5%25BA%258F\\%25E7%25BB%2593%25E6%259E%259C%25E5%259B%25BE%25E7%2589%2587\\GO%25E5%2588%2586%25E6%259E%2590\\N15_vs_NT.P.html#gene11) | single-organism cellular process | 137 (67.49%) | 928 (54.56%) | 0.000046 | 0.000659 |
| [GO:0065007](file:///E:\\000000CAR%20NK\\00000%25E6%25AF%2595%25E4%25B8%259A%25E8%25AE%25BA%25E6%2596%2587\\%25E5%25AE%259E%25E9%25AA%258C%25E7%25BB%2593%25E6%259E%259CPPT%25E6%25B1%2587%25E6%2580%25BB\\9%20%25E8%25BD%25AC%25E5%25BD%2595%25E7%25BB%2584%25E6%25B5%258B%25E5%25BA%258F\\%25E7%25BB%2593%25E6%259E%259C%25E5%259B%25BE%25E7%2589%2587\\GO%25E5%2588%2586%25E6%259E%2590\\N15_vs_NT.P.html#gene12) | biological regulation | 98 (48.28%) | 607 (35.68%) | 0.000059 | 0.000772 |
| [GO:0007154](file:///E:\\000000CAR%20NK\\00000%25E6%25AF%2595%25E4%25B8%259A%25E8%25AE%25BA%25E6%2596%2587\\%25E5%25AE%259E%25E9%25AA%258C%25E7%25BB%2593%25E6%259E%259CPPT%25E6%25B1%2587%25E6%2580%25BB\\9%20%25E8%25BD%25AC%25E5%25BD%2595%25E7%25BB%2584%25E6%25B5%258B%25E5%25BA%258F\\%25E7%25BB%2593%25E6%259E%259C%25E5%259B%25BE%25E7%2589%2587\\GO%25E5%2588%2586%25E6%259E%2590\\N15_vs_NT.P.html#gene13) | cell communication | 96 (47.29%) | 596 (35.04%) | 0.000085 | 0.000896 |
| [GO:0023052](file:///E:\\000000CAR%20NK\\00000%25E6%25AF%2595%25E4%25B8%259A%25E8%25AE%25BA%25E6%2596%2587\\%25E5%25AE%259E%25E9%25AA%258C%25E7%25BB%2593%25E6%259E%259CPPT%25E6%25B1%2587%25E6%2580%25BB\\9%20%25E8%25BD%25AC%25E5%25BD%2595%25E7%25BB%2584%25E6%25B5%258B%25E5%25BA%258F\\%25E7%25BB%2593%25E6%259E%259C%25E5%259B%25BE%25E7%2589%2587\\GO%25E5%2588%2586%25E6%259E%2590\\N15_vs_NT.P.html#gene14) | signaling | 96 (47.29%) | 596 (35.04%) | 0.000085 | 0.000896 |
| [GO:0044700](file:///E:\\000000CAR%20NK\\00000%25E6%25AF%2595%25E4%25B8%259A%25E8%25AE%25BA%25E6%2596%2587\\%25E5%25AE%259E%25E9%25AA%258C%25E7%25BB%2593%25E6%259E%259CPPT%25E6%25B1%2587%25E6%2580%25BB\\9%20%25E8%25BD%25AC%25E5%25BD%2595%25E7%25BB%2584%25E6%25B5%258B%25E5%25BA%258F\\%25E7%25BB%2593%25E6%259E%259C%25E5%259B%25BE%25E7%2589%2587\\GO%25E5%2588%2586%25E6%259E%2590\\N15_vs_NT.P.html#gene15) | single organism signaling | 96 (47.29%) | 596 (35.04%) | 0.000085 | 0.000896 |

# Supplementary Figures.

**Supplementary Figure 1. Identification of B-LCL lines**

CD19 expression of PBMC and B-LCL detected by flow cytometry. The CD19^+^ percentage of PBMC was 8.46±2.40% from 4 individual blood donors, while that in the B-LCL was 97.67± 1.45%. This indicates that after EBV infection, B lymphocytes successfully transform into B-LCL.

**A B**

**Supplementary Figure 2. Schematic diagram of the pSFG-NKG2D CAR and pSFG-IL-15/IL-15Rα-NKG2D CAR plasmids**

(**A**) pSFG-NKG2D CAR plasmid map (CAR: 1142bp); (**B**) pSFG-IL-15/IL-15Rα-NKG2D CAR plasmid map (CAR: 1913bp). Ncol I and Mlu I restriction sites in the pSFG vector for the insert fragment were shown.

**Supplementary Figure 3. Identification of pSFG-NKG2D CAR and pSFG-IL-15/IL-15Rα-NKG2D CAR by restriction enzyme digestion and Sanger sequencing**

(**A**) Identification of pSFG-NKG2D CAR by restriction enzyme digestion, M: 10000 DNA Marker; 1: Plasmid DNA (8818bp); 2: SphI single restriction enzyme digestion (8607bp/404bp); 3: NcoI+MluI double enzyme digestion (7676bp/1142bp). (**B**) Identification of pSFG-IL-15/IL-15Rα-NKG2D CAR-T by restriction enzyme digestion, M: 10000 DNA Marker; 1: Plasmid DNA (9589bp); 2: SphI single restriction enzyme digestion (8039bp/1149bp/404bp); 3: NcoI+MluI double enzyme digestion (7676bp/1913bp). C&D. Sanger sequencing of NKG2D CAR and IL-15/IL-15Rα-NKG2D CAR.

**Supplementary Figure 4. pSFG-NKG2D CAR and pSFG-IL-15/IL-15Rα-NKG2D CAR plasmid expression in 293T cells detected by Western blot**

(**A**) Transduction of pSFG vector to 293T cells (eGFP^+^, by inverted fluorescence microscope, scale=400μm). After transfecting N and N15 groups with the SFG-CAR plasmid for 72h, the cell growth confluence rate was observed to be 90% with GFP expression ranging from 70% to 80% under a fluorescence microscope. (**B**) Identification of NKG2D CAR and IL-15/IL-15Rα expressions in the precipitates and supernatants of 293T cell cultures by Western blot after transduction with three types of retroviruses. N: NKG2D CAR retrovirus transduction; N15: IL-15/IL-15Rα-NKG2D CAR retrovirus transduction; Ctrl: empty retrovirus transduction. The correct bands of NKG2D CAR fragment (1142bp, 43kDa) present in the cell precipitates of N and N15 groups, while no band was observed in Ctrl group. The protein expression level in N15 group was lower than that in N group, which may be attributed to the larger fragment carried by the plasmid. The IL-15/IL-15Rα sequence utilized in this research comprises three components: IL-15 (342bp), a linker peptide (60bp), and the Sushi domain of IL-15Rα (a high-affinity IL-15 binding region, 285bp), with the addition of a His-tag at the sequence terminus. The complete protein size is approximately 25kDa. Detection using anti-His-tag and anti-IL-15 antibodies revealed double bands in both cell precipitate and supernatant of N15 group, consistent with theoretical values. The appearance of double bands may be attributed to the existence of two distinct structures for IL-15/IL-15Rα, which remain unchanged during protein denaturation.

**Supplementary Figure 5. Gating strategy of Flow cytometry Absolute Counting**

(**A**) Set up FSC-SSC gate, circle the counting beads and lymphocyte population; then circle the live cell population from lymphocyte population; finally, calculate the live cell population according the formula mentioned in Supplementary methods. (**B, C, D**). Gating examples of NT, N and N15 groups, respectively.


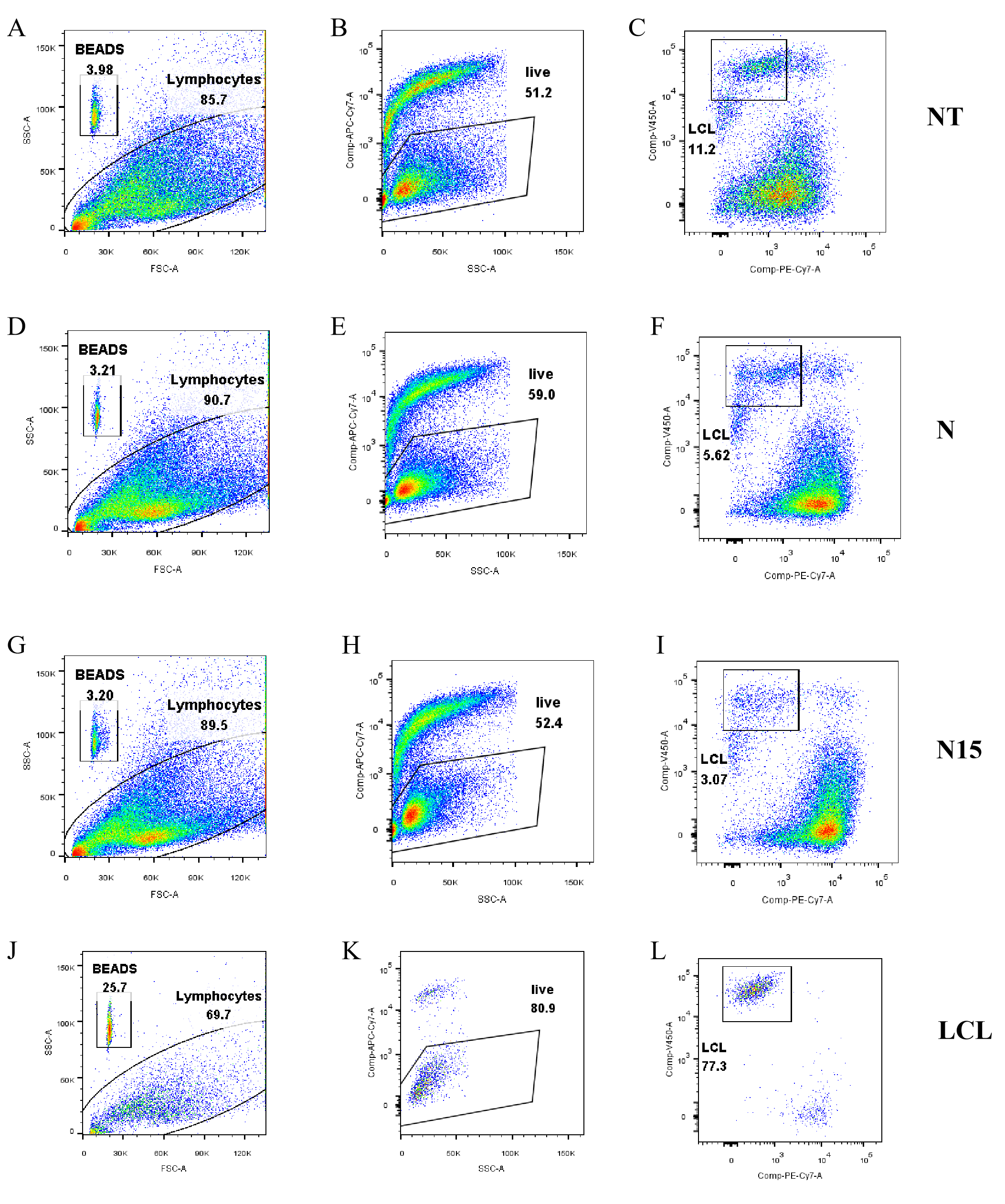


**Supplementary Figure 6. Gating strategy in flow cytometric cytotoxicity assay for the co-cultures of B-LCLs and three types of T cells**.

(**A**-**C**) NT-T cell group. (**D**-**F**) N CAR-T cell group. (**G**-**I**). N15 CAR-T cell group. First column (**A**,**D**,**G**,**J**): Adjust the voltages of forward scattering (FSC) and side scattering (SSC) to make the counting beads locate in the appropriate position; Circle out the counting beads and collect 2000 bead events. Second column (**B**,**E**,**H**,**K**): Circle out the live cell population from lymphocyte population (APC-Cy 7-). Third column (**C**,**F**,**I**,**L**): Circle out the B-LCL population (Cell Trace V450). According to the value of beads events and B-LCL events, the absolute count of B-LCLs is calculated; then the percentage of target cell lysis is calculated with B-LCL count in co-culture well and target cell alone well according to Formula I.

**Supplementary Figure 7．Correlation of EGFP to NKG2D expression in three types of T cells**.

(**A**) Representative of flow cytometric scatter plot of EGFP and NKG2D expressions of NT-T, N and N15 CAR-T cell groups on Day 13 post transduction. (**B**) Statistic graph of NKG2D ligand expression in B-LCLs and PBMCs the EGFP and NKG2D expressions of NT-T, N and N15 CAR-T cell groups on Day 13 post transduction.

**Supplementary Figure 8．The subsets of CD4/CD8, specific activation (CD25 and CD69) and exhaustion markers (PD-1, LAG-3 and TIM-3) detected by flow cytometry**.

(**A**) Flow cytometric scatter plot of T cell subsets (CD8/CD4). (**B**) Ratio of CD8^+^/CD4^+^ T cells. (**C**) Flow cytometric histograms of CD259 expression in CD3^+^ NT-T, N and N15 CAR-T cells after CAR transduction. (**D**) Statistics graph of CD25 expression. (**E**) Flow cytometric histograms of CD69 expression in CD3^+^ NT-T, N and N15 CAR-T cells after CAR transduction. (**F**). Statistics graph of CD69 expression. (**G**,**I**,**K**). Flow cytometric analysis graph (Zeta Plot) of PD-1, LAG-3 and TIM-3 expression in CD3^+^ T cells. (**H**,**J**,**L**). Statistics graph of PD-1, LAG-3 and TIM-3 expression in CD3^+^ T cells. * *P* <0.05； ** *P* <0.01 ； *** *P* <0.001；**** *P* <0.0001.


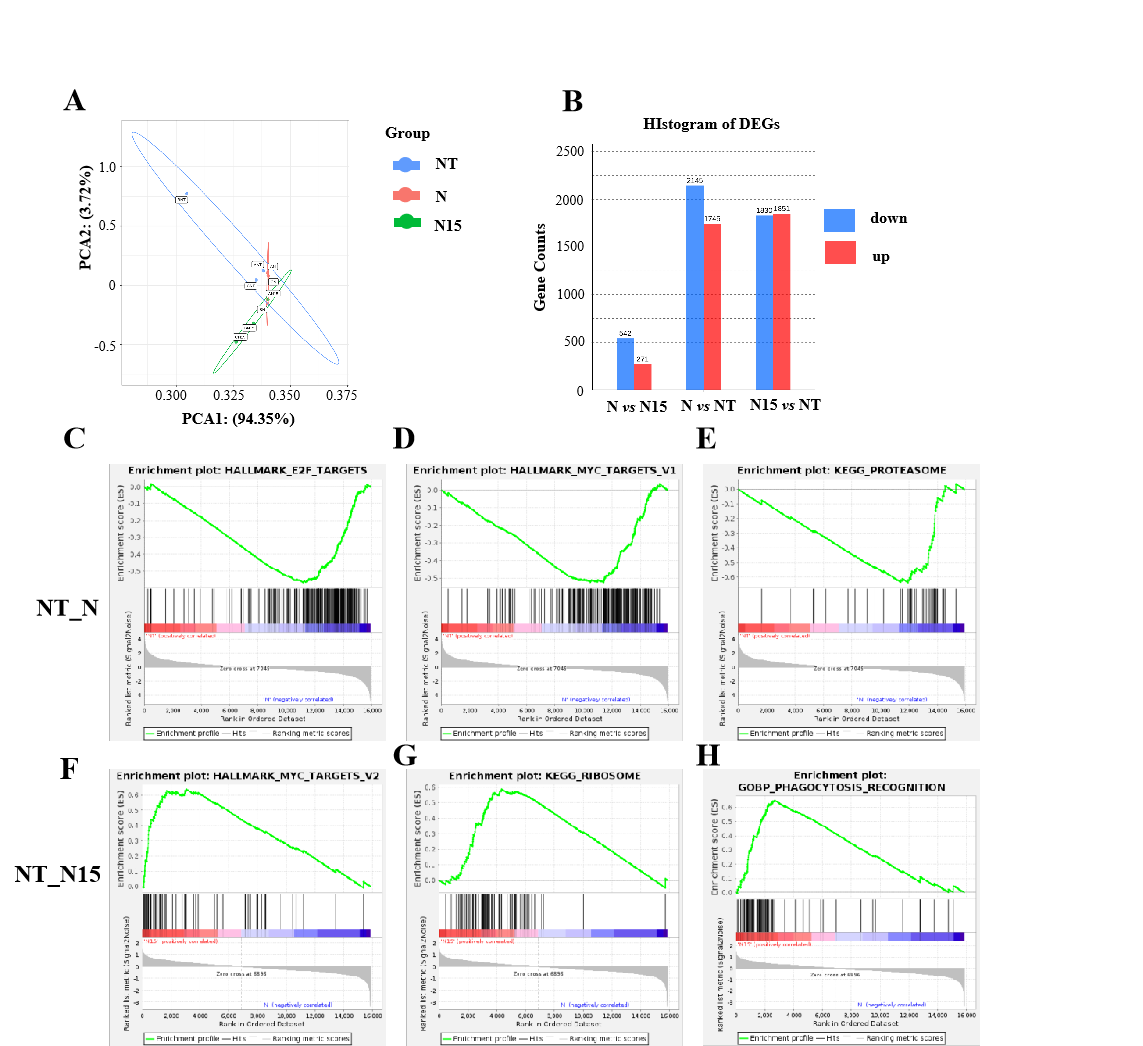


**Supplementary Figure 9. Analysis of transcriptomes from three types of T cells.**

(**A**) PCA analysis. (**B**) Statistical chart of DEGs. (**C**-**H**) Related pathways identified by GSEA pathway enrichment.

**Supplementary Figure 10. Measurement of secretion cytokines in 6-72 h co-cultures of B-LCLs with three types of T cells by ELISA**.

(**A**) IL-15. (**B**) IL-2. (**C**) IL-10. (**D**) IFN-γ. (**E**) GM-CSF. (**F**) TNF-α. (**G**) IL-6. (n=5) * *P* <0.05; ** *P* <0.01; *** *P* <0.001; **** *P* <0.0001.

**Supplementary Figure 11. Representatives of flow cytometric plot for measuring B-LCLs, NT-T, N and N15 CAR-T cells from the treated B-LCL mouse models**.

(**A**) The proportion of B-LCLs in liver. (**B**) The proportion of T cells in spleen. (**C**) The proportion of EGFP^+^ CAR-T cells in spleen. * *P*<0.05; ** *P*<0.01; *** *P*<0.001; **** *P*<0.0001.
